# A *Myb* enhancer-guided analysis of basophil and mast cell differentiation

**DOI:** 10.1038/s41467-022-34906-1

**Published:** 2022-11-18

**Authors:** Takayoshi Matsumura, Haruhito Totani, Yoshitaka Gunji, Masahiro Fukuda, Rui Yokomori, Jianwen Deng, Malini Rethnam, Chong Yang, Tze King Tan, Tadayoshi Karasawa, Kazuomi Kario, Masafumi Takahashi, Motomi Osato, Takaomi Sanda, Toshio Suda

**Affiliations:** 1 Cancer Science Institute of Singapore, National University of Singapore, Singapore, Singapore; 2 Division of Inflammation Research, Center for Molecular Medicine, Jichi Medical University, Tochigi, Japan; 3 Division of Cardiovascular Medicine, Department of Medicine, Jichi Medical University School of Medicine, Tochigi, Japan; 4 Signature Program in Neuroscience and Behavioral Disorders, Duke-NUS Medical School, Singapore, Singapore; 5 International Research Center for Medical Sciences, Kumamoto University, Kumamoto, Japan; 6 Department of Medicine, Yong Loo Lin School of Medicine, National University of Singapore, Singapore, Singapore

**Keywords:** Haematopoietic stem cells, Myelopoiesis, Myelopoiesis, Immunological surveillance, Bone marrow

## Abstract

The transcription factor MYB is a crucial regulator of hematopoietic stem and progenitor cells. However, the nature of lineage-specific enhancer usage of the *Myb* gene is largely unknown. We identify the *Myb* −68 enhancer, a regulatory element which marks basophils and mast cells. Using the *Myb* −68 enhancer activity, we show a population of granulocyte-macrophage progenitors with higher potential to differentiate into basophils and mast cells. Single cell RNA-seq demonstrates the differentiation trajectory is continuous from progenitors to mature basophils in vivo, characterizes bone marrow cells with a gene signature of mast cells, and identifies LILRB4 as a surface marker of basophil maturation. Together, our study leads to a better understanding of how MYB expression is regulated in a lineage-associated manner, and also shows how a combination of lineage-related reporter mice and single-cell transcriptomics can overcome the rarity of target cells and enhance our understanding of gene expression programs that control cell differentiation in vivo.

## Abstract (web summary)

The transcription factor MYB has been shown to regulate haematopoietic stem cells but there could be lineage specific enhancers. Here, using lineage tracing and single cell sequencing the authors characterise a *Myb* −68 enhancer that regulates the differentiation of mast cells and basophils.

## Introduction

Gene expression is regulated by a network of transcription factors that bind to cis-regulatory sequences including promoters and enhancers, and their diversity is now considered to be central to distinct expression of each gene^1–5^. Multiple lines of evidence has shown that enhancers are occupied with active histone marks in a cell type-specific manner while promoters are more ubiquitously marked among various cell types. Genes expressed broadly among multi-lineages can show a vast difference in enhancer usage across distinct differentiation stages, suggesting that enhancers control appropriate spatio-temporal gene expression more than promoters^6,7^. Further, during blood cell differentiation, it is indicated that enhancer establishment is initiated in early lineage commitment and can dictate the differentiation potential of progeny before the execution of the RNA expression program^8^.

The transcription factor MYB is a key regulator of hematopoietic stem and progenitor cells (HSPC)^9,10^, and required for various stages of hematopoietic differentiation, including hematopoietic stem cell (HSC) self-renewal, myeloid progenitors development^11,12^, erythropoiesis^13^, B cell differentiation^14^, and T cell development^15^. It is also shown that appropriate levels of MYB expression are required at distinct differentiation steps of each hematopoietic cell lineage^16^. However, little is understood about how MYB expression is regulated in each of hematopoietic sub-lineages.

In this work, to gain insight into lineage-specific control of MYB expression, we identify 2 lineage-related enhancers of *Myb* by combining in silico screening and in vivo validation using zebrafish and transgenic mice. One is lymphoid lineage-oriented, and the other is myeloid lineage-oriented, and both enhancers have activities associated to the specific subsets of hematopoietic cell differentiation. We confirm that the *Myb* −68 enhancer is an enhancer which marks basophils and mast cells. Further, *Myb* −68 enhancer-guided single cell analysis indicates that the differentiation trajectory is continuous from progenitors to mature basophils in vivo, providing a reference for future studies.

## Results

### In silico analysis and in vivo validation using zebrafish identified cis-regulatory elements of *Myb*

**Fig. 1:**
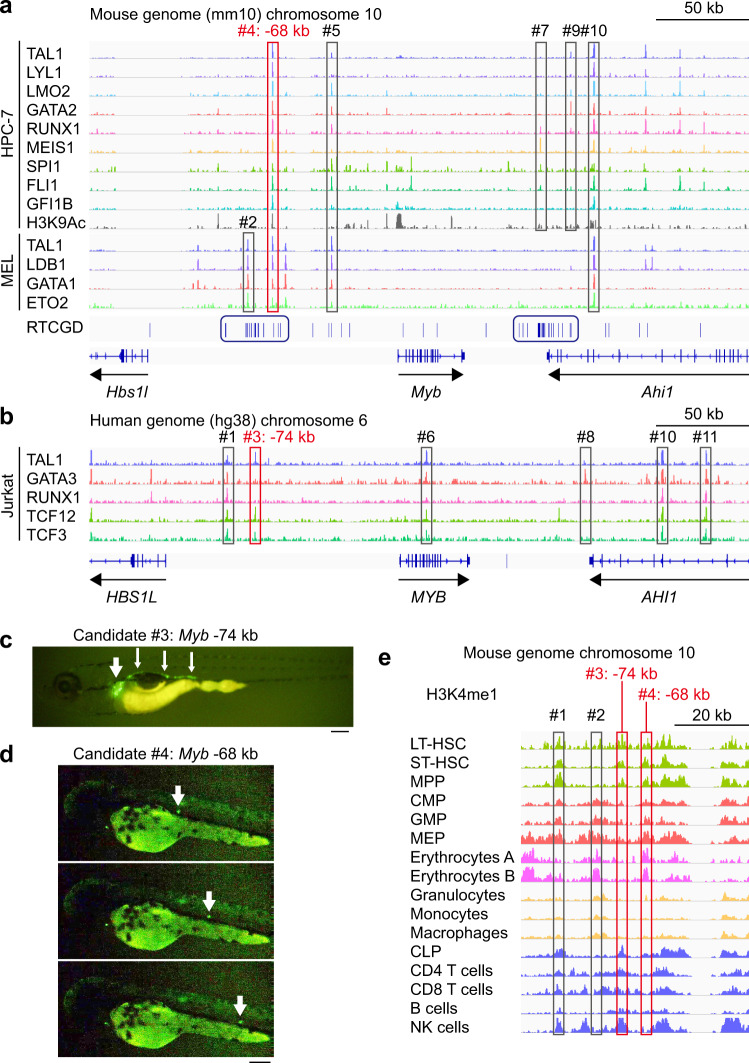
In silico analysis and in vivo validation using zebrafish to identify cis-regulatory elements of *Myb*. **a**, **b** Analysis of published ChIP-seq data demonstrates that multiple transcription factors bind around the mouse *Myb* locus in HPC-7 cells (**a**, upper) and MEL cells (**a**, lower) and the human *MYB* locus in Jurkat cells (**b**). A total of 11 candidate enhancer elements are shown in red or grey rectangles (#1 to #11). Elements #4 and #5 are common between HPC-7 cells and MEL cells, and the element #10 is common among 3 cell lines. Two clusters of retroviral integration sites (RIS) are indicated in blue rectangles. RTCGD Retrovirus Tagged Cancer Gene Database. **c**, **d** In vivo functional confirmation of selected regions using zebrafish. The candidate cis-regulatory elements were cloned into the upstream of an EGFP reporter construct, and injected into zebrafish embryos. **c** The EGFP activity driven by the candidate element #3 (*Myb* −74 kb) is specific to zebrafish hematopoietic organs: kidneys (pronephros, white thick arrow) and pronephric tubules (white thin arrows). Scale bar: 0.2 mm. **d** The EGFP activity by the candidate element #4 (*Myb* −68 kb) is specific to circulating cells (white arrows). Three snapshots from one video are shown. Scale bar: 0.2 mm. **e** Analysis of publicly available ChIP-seq data demonstrates that H3K4me1 signal intensity is relatively high in the candidate element #3 (*Myb* −74 kb) of hematopoietic stem/progenitor cells and lymphoid cells, and in the candidate element #4 (*Myb* −68 kb) of hematopoietic stem/progenitor cells and myeloid cells.

Retroviral insertional mutagenesis has been used as a potent cancer gene discovery tool, and mapping of common retroviral integration sites (RIS) which represent open accessible chromatin provides molecular tags to identify potential regulatory elements for subsequent experimental verification^17,18^. To discover cis-regulatory elements of *Myb* which act in a lineage-specific manner, we first selected two clusters of RISs around the *Myb* locus, using the Retrovirus Tagged Cancer Gene Database (Fig. 1a). Next, we took advantage of three published ChIP-seq datasets to narrow down the regions bound by hematopoietic transcription factors: ChIP-seq data of mouse hematopoietic progenitor HPC-7 cells^19^, mouse erythroleukaemia MEL cells^20^ (Fig. 1a), and human T-cell acute lymphoblastic leukemia (T-ALL) Jurkat cells^21^ (Fig. 1b). Of note, the binding profile of the TAL1 complex is largely different among these cell types, highlighting the differential usage of lineage-specific enhancers. Based on these data, we selected a total of 11 candidate regulatory elements for further in vivo validation (Supplementary Table 1).

We then validated the activity of each element in vivo using zebrafish. Each of candidate elements was cloned into enhanced green fluorescent protein (EGFP) reporter constructs, and injected into one-cell stage zebrafish embryos. While most elements failed to drive tissue-specific EGFP expression, the *Myb* -74 kb element (#3) reproducibly induced precise EGFP expression at hematopoietic sites in zebrafish, namely kidneys (pronephros) and pronephric tubules^22^ (Fig. 1c), and the *Myb* -68 kb element (#4) led to EGFP expression only in circulating cells within the blood vessels (Fig. 1d), confirming the in vivo regulatory potential of these regions in hematopoiesis. Both regions contain multiple transcription factor binding motifs conserved among species (Supplementary Fig. 1a, b). Additional in silico analysis using ChIP-seq datasets from mouse HSCs to differentiated cells for histone H3 lysine 4 monomethylation (H3K4me1)^8^ suggested that these two elements possessed different enhancer activity among cell types: the *Myb* -74 kb element mainly in HSPCs and lymphoid cells, and the *Myb* -68 kb element mainly in HSPCs and myeloid cells (Fig. 1e).

We also re-analyzed our previously published histone H3 lysine 27 acetylation (H3K27Ac) Hi-ChIP data obtained from mouse T-ALL cells^23^, and confirmed looping between the *Myb* -74 kb element and the *Myb* promoter (Supplementary Fig. 1c). The *Myb* -68 kb element was also previously shown to interact with the *Myb* promoter in MEL cells^20^.

Taken together, the *Myb* -74 kb element and the *Myb* -68 kb element (hereafter referred to as *Myb* -74 enhancer and *Myb* -68 enhancer, respectively) appeared to be lineage-dependent enhancers of the *Myb* gene.

### The *Myb* -74 enhancer is activated robustly in immature T and B cells

To further explore in vivo biological significance of identified enhancers, we designed EGFP reporter constructs containing either the *Myb* -74 or -68 enhancer located upstream of the mouse heatshock protein 68 minimal promoter (mhsp68p), and then transgenic reporter mice carrying these reporter constructs were generated (hereafter referred to as *Myb* -74 GFP mice and *Myb* -68 GFP mice, respectively) (Supplementary Fig. 2a).

In bone marrow cells of *Myb* -74 GFP mice, GFP activity was detected only in subsets of B cells and T cells (Supplementary Fig. 2b). Interestingly, Lin^-^Sca1^+^cKit^+^ (LSK) and Lin^-^Sca1^-^cKit^+^ (LS-K) progenitor cells, which were known to have higher endogenous MYB expression than differentiated cells, were not marked by GFP, indicating that the *Myb* -74 enhancer did not recapitulate the wider expression of endogenous MYB. Because the *Myb* -74 enhancer was originally defined by ChIP-seq data of Jurkat cells, these findings are consistent with the hypothesis that this enhancer is lymphoid-oriented.

In-depth analysis of the B cell lineage showed that GFP activity driven by the *Myb* -74 enhancer turned on in B220^low^CD24^-^Ly-51^-^ Pre-Pro-B cells, and then attenuated along B cell maturation (Supplementary Fig. 2c). Loss of MYB is known to cause a partial block during B cell development at the Pro-B to Pre-B cell transition^14^. We speculate that the *Myb* -74 enhancer activity is required to turn on at the Pre-Pro-B cell stage to induce sufficient expression of *Myb* at the next differentiation stage.

The thymus of *Myb* -74 GFP mice showed robust GFP activity after the early T lineage progenitor (ETP) stage, and two activity peaks along T cell differentiation were observed: one in the double negative 3 (DN3) stage and the other in the CD4/CD8 single positive (SP) stage (Supplementary Fig. 2d). This is also likely to reflect known functions of MYB at multiple checkpoints during thymocyte development including the transition through DN3 stage, survival of double positive (DP) cells and CD4 SP cells differentiation^15^. Splenic T cells, especially CD4^+^CD44^+^CD62L^-^ memory T cells, had reduced GFP activity, suggesting that GFP activity declined with T cell maturation, similarly to B cell maturation.

### The *Myb* -68 enhancer activity is induced in granulocyte/macrophage-lineage progenitors

**Fig. 2:**
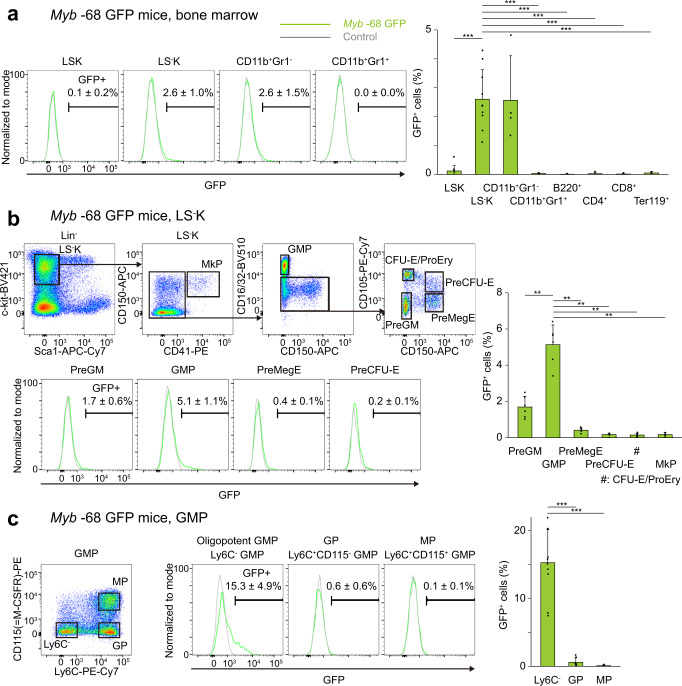
The *Myb* -68 enhancer is active in Ly6C^-^ GMPs in *Myb* -68 GFP transgenic mice. GFP activity in whole bone marrow (**a**), LS^-^K cells (**b**), and GMPs (**c**) of *Myb* -68 GFP mice. FACS gating strategies are shown in Supplementary Fig. 12. Representative FACS plots of indicated cells from control (grey) and *Myb* -74 GFP mice (green) are shown. Numbers shown are mean percentages±SD of GFP^+^ cells in *Myb* -68 GFP mice. The percentages of GFP^+^ cells are also shown in the right graphs. **a** *n* = 10 mice for LSK cells and LS^-^K cells, and *n* = 4 mice for others. *p* = 4.1 × 10^−^^4^ by one-way Welch’s ANOVA. ^***^p < 0.001 by the Games-Howell post hoc test. *p* = 4.1 × 10^−^^4^ between LSK and LS^-^K, *p* = 3.7 x 10^−^^4^ between CD11b^+^Gr1^+^ and LS^-^K, *p* = 3.5 x 10^−^^4^ between B220^+^ and LS^-^K, *p* = 3.5 x 10^−^^4^ between CD4^+^ and LS^-^K, *p* = 3.5 x 10^−^^4^ between CD8^+^ and LS^-^K, and *p* = 3.9 × 10^−^^4^ between Ter119^+-^ and LS^-^K. (**b**) *n* = 6 mice. *p* = 5.8 × 10^−^^7^ by one-way Welch’s ANOVA. ^**^p < 0.01 by the Games-Howell post hoc test. *p* = 0.0014 between PreGM and GMP, *p* = 0.0008 between PreMegE and GMP, p = 0.0007 between PreCFU-E and GMP, *p* = 0.0006 between CFU-E/ProEry and GMP, and p = 0.0006 between MkP and GMP. **c** *n* = 10 mice for Ly6C^-^ GMPs and *n* = 7 mice for others. *p* = 8.5 × 10^−^^6^ by one-way Welch’s ANOVA. ^***^p < 0.001 by the Games-Howell post hoc test. *p* = 1.1 × 10^−^^5^ between Ly6C^-^ GMPs and GP, and *p* = 1.1 × 10^−^^5^ between Ly6C^-^ GMPs and MP. LSK Lin^-^Sca1^+^cKit^+^ cells, LS^-^K Lin^-^Sca1^-^cKit^+^ cells, MkP megakaryocyte progenitor, GMP granulocyte/monocyte progenitor, PreGM pre-granulocyte/macrophage, CFU-E colony-forming unit-erythroid, ProEry pro-erythrocytes, PreCFU-E pre-colony-forming unit-erythroid, PreMegE pre-megakaryocyte/erythrocyte, GP granulocyte progenitor, MP monocyte progenitor.

In contrast to *Myb* -74 GFP mice, *Myb* -68 GFP mice showed GFP activity only in a very small percentage of LS-K progenitor cells and CD11b^+^Gr1^-^ myeloid cells (Fig. 2a and Supplementary Figs. 3a, b). Further analysis of LS-K progenitor cells demonstrated that GFP activity was found only in subsets of LS-KCD41^-^FcγR^-^CD150^-^CD105^-^ pre-granulocyte/macrophage (Pre-GM) cells and LS-KCD41^-^FcγR^+^CD150^-^ granulocyte-macrophage progenitors (GMP)^24^ (Fig. 2b). This is in stark contrast to uniformly high expression of endogenous MYB among all subsets of myeloid progenitors^12^.

In general, GMPs are subdivided into oligopotent Ly6C^-^ GMPs and their lineage-committed progeny, Ly6C^+^CD115^-^ granulocyte progenitors (GP) and Ly6C^+^CD115^+^ monocyte progenitors (MP)^25^. Interestingly, GFP activity was further enriched only in oligopotent Ly6C^-^ GMPs while lineage-committed GPs and MPs showed no GFP activity (Fig. 2c). This result suggested that the *Myb* -68 enhancer could be active in specific myeloid sub-lineages other than granulocytes or monocytes.

### The *Myb* -68 enhancer function persists only in basophils and mast cells

**Fig. 3:**
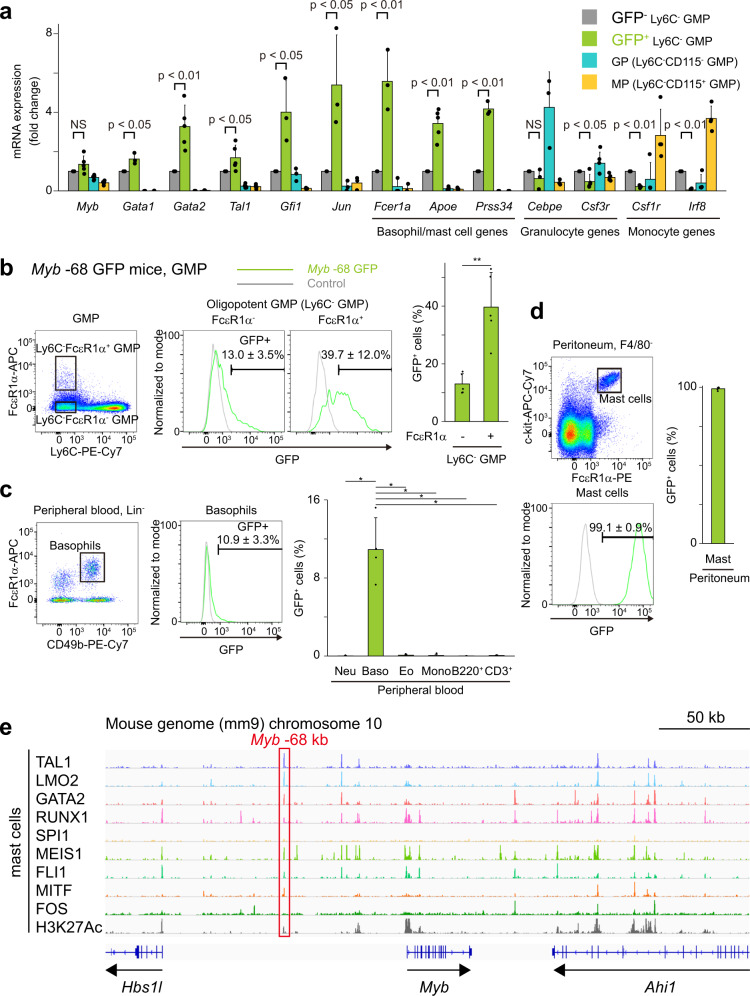
The *Myb* -68 enhancer function persists only in basophils and mast cells. **a** mRNA expression levels of stem/progenitor and lineage marker genes in GFP^-^Ly6C^-^ GMPs (grey), GFP^+^Ly6C^-^ GMPs (green), granulocyte progenitors (GP, blue) and monocyte progenitors (MP, yellow) (*n* = 5 mice for *Myb*, *Gata2*, *Tal1*, *Apoe*, and *Csf3r*, *n* = 4 mice for *Csf1r* and *Irf8*, and *n* = 3 mice for others). FACS gating strategies are shown in Supplementary Fig. 12 and Fig. 2c. Data are presented as mean values ± SD. *p* = 0.10, 0.018, 0.0016, 0.044, 0.032, 0.040, 0.0066, 8.9 × 10^−^^5^, 9.3 × 10^−^^5^, 0.27, 0.013, 2.8 × 10^−^^7^, and 1.0 × 10^−^^7^ from left to right by two-tailed Student’s *t*-test without adjustments for multiple comparisons. GFP activity in FcεR1α^-^ and FcεR1α^+^ Ly6C^-^ GMPs (**b**), basophils in peripheral blood (**c**), and mast cells in the peritoneal cavity (**d**) of *Myb* -68 GFP mice. FACS gating strategies are shown in Supplementary Fig. 12, 13c and 13d. Representative FACS plots of indicated cells from control (grey) and *Myb* -74 GFP mice (green) are shown. Numbers shown are mean percentages±SD of GFP^+^ cells in *Myb* −68 GFP mice. The percentages of GFP^+^ cells are also shown in the right graphs. Data are presented as mean values±SD. **b** *n* = 5 mice. ^**^p = 0.0014 by two-tailed Student’s *t*-test. **c** Lin^-^ indicates CD4^-^CD8^-^B220^-^Gr1^-^Ly6C^-^CD11c^-^cKit^-^Ter119^-^SiglecF^-^IL7Ra^-^NK1.1^-^. *n* = 4 mice for basophils and *n* = 5 mice for others. *p* = 0.0041 by one-way Welch’s ANOVA. ^*^p < 0.05 by the Games-Howell post hoc test. *p* = 0.032 between neutrophils (Neu) and basophils (Baso), *p* = 0.033 between eosinophils (Eo) and Baso, *p* = 0.033 between monocytes (Mono) and Baso, *p* = 0.032 between B220^+^ cells and Baso, and *p* = 0.033 between CD3^+^ cells and Baso. **d** *n* = 5 mice. **e** Analysis of published ChIP-seq data demonstrates that multiple transcription factors including MITF bind to the *Myb* -68 enhancer (red rectangle).

To elucidate the nature of GFP^+^Ly6C^-^ GMPs, mRNA expression levels of HSPC and lineage marker genes were compared among GFP^-^Ly6C^-^ GMPs, GFP^+^Ly6C^-^ GMPs, GPs and MPs (Fig. 3a). While monocyte-lineage markers, *Csf1r* and *Irf8*, and a granulocyte-lineage marker *Csfr3* were downregulated, *Gata1*, *Gata2*, *Tal1*, *Gfi1*, and *Jun* were significantly higher in GFP^+^Ly6C^-^ GMPs than in GFP^-^Ly6C^-^ GMPs. Because GATA2 in GMPs is critical for basophil and mast cell differentiation^26,27^, higher *Gata2* expression in GFP^+^Ly6C^-^ GMPs prompted us to examine whether these cells were related to basophil and mast cell differentiation. As expected, basophil/mast cell markers, *Fcer1a*, *Apoe*, and *Prss34*^28^ were highly induced in GFP^+^Ly6C^-^ GMPs. Of note, *Myb* mRNA expression showed no difference between GFP^-^Ly6C^-^ GMPs and GFP^+^Ly6C^-^ GMPs, demonstrating another example of the discrepancy between the enhancer specificity and the promoter specificity.

In line with gene expression data, surface expression of the IgE receptor FcεR1α further enriched GFP^+^ cells in GFP^+^Ly6C^-^ GMPs (Fig. 3b). In peripheral blood of *Myb* -68 GFP mice, basophils, but not neutrophils, monocytes, and lymphocytes, showed GFP activity, albeit at a lower level, and mast cells in the peritoneal cavity showed the highest GFP activity (Figs. 3c, d). Collectively, the *Myb* -68 enhancer is basophil/mast cell-associated, and its activity is turned on at the GMP stage, persisted during basophil differentiation and further increased during mast cell differentiation. Analysis of published mRNA expression datasets also indicated that *MYB* mRNA expression in basophils was the highest among differentiated cells in human peripheral blood (Supplementary Fig. 3c), and that mouse mast cells had even higher expression of *Myb* than basophils (Supplementary Fig. 3d), demonstrating that the *Myb* -68 enhancer activity and *Myb* mRNA expression were correlated to some extent among differentiated cells.

Furthermore, analysis using ChIP-seq datasets from bone marrow-derived mast cells^29^ showed that not only multiple HSC-related transcription factors but also the key mast cell regulator MITF bound to the *Myb* -68 enhancer (Fig. 3e), suggesting the activity of the *Myb* -68 enhancer driven by MITF in mast cells.

### The *Myb* -68 enhancer can enrich basophil/mast cell-biased GMPs before FcεR1α surface expression

**Fig. 4:**
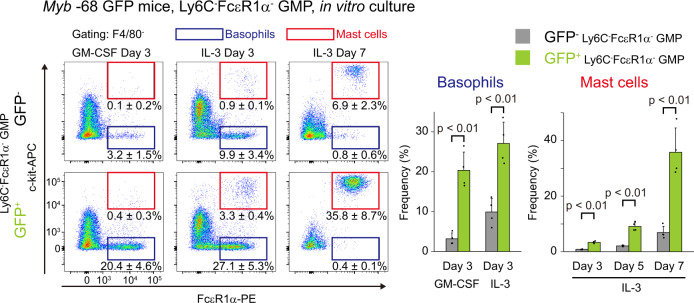
The *Myb* -68 enhancer enriches basophil/mast cell-biased GMPs before FcεR1α surface expression. Representative FACS plots of GFP^-^ (upper) and GFP^+^ (lower) Ly6C^-^FcεR1α^-^ GMPs cultured in vitro in the presence of GM-CSF or IL-3 for indicated periods of time. FACS gating strategies are shown in Supplementary Figure 14a. Numbers shown are mean percentages±SD of basophils, defined as FcεR1α^+^c-kit^-^ (blue), and mast cells, defined as FcεR1α^+^c-kit^+^ (red). The percentages of basophils and mast cells derived from GFP^-^ (grey) and GFP^+^ (green) Ly6C^-^FcεR1α^-^ GMPs are also shown in the right graph (*n* = 4 biological replicates from 2 independent experiments). *p* = 3.8 × 10^−^^4^, 1.6 × 10^−^^3^, 2.3 × 10^−^^5^, 1.0  × 10^−^^4^, and 6.9 × 10^-4^ from left to right by two-tailed Student’s *t*-test without adjustments for multiple comparisons.

Because the *Myb* -68 enhancer activity in FcεR1α^-^Ly6C^-^ GMPs precedes FcεR1α surface expression, next we investigated whether the *Myb* -68 enhancer could enrich basophil/mast cell-biased GMPs before the onset of FcεR1α surface expression (Fig. 4). FACS-sorted GFP^-^ and GFP^+^ FcεR1α^-^Ly6C^-^ GMPs were cultured with GM-CSF or IL-3. Indeed after 3 days under both culture conditions, GFP^+^ FcεR1α^-^Ly6C^-^ GMPs gave rise to more basophils than GFP^-^ FcεR1α^-^Ly6C^-^ GMPs. In the presence of IL-3, GFP^+^ FcεR1α^-^Ly6C^-^ GMPs differentiated more into mast cells consistently during 7 days culture. These findings confirmed that the *Myb* -68 enhancer activity enriched GMPs with the high capacity to differentiate into basophils and mast cells before FcεR1α surface expression.

### The *Myb* -68 enhancer regulates *Myb* mRNA expression and basophil and mast cell differentiation

**Fig. 5:**
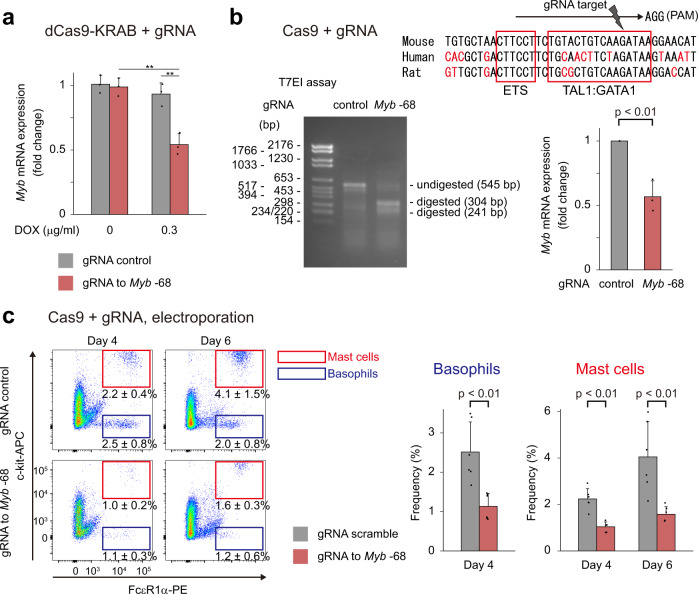
The *Myb* -68 enhancer regulates *Myb* mRNA expression and basophil and mast cell differentiation. **a** Mouse mast cell P815 cells were infected with lentivirus harboring dCas9-KRAB and DOX-inducible control gRNA (grey) or gRNA targeted for the *Myb* -68 enhancer (red), sorted and treated with doxycycline (0.3 ng/ml) for 72 hours. *n* = 3 independent experiments. Data are presented as mean values±SD. *p* = 2.4 × 10^−^^4^ by one-way ANOVA. ^**^p < 0.01 by the Tukey-Kramer post hoc test. *p* = 5.0 × 10^−^^4^ and 1.2 × 10^−^^3^ from left to right. **b** P815 cells were infected with lentivirus harboring Cas9 and DOX-inducible control gRNA (grey) or gRNA targeted for the *Myb* -68 enhancer (red). Cells were sorted and treated with doxycycline (1 ng/ml) for 72 h. Genome editing efficiency was evaluated by T7EI mismatch detection assay (left), and *Myb* mRNA expression was analyzed by qPCR (right). *n* = 3 independent experiments. Data are presented as mean values±SD. *p* = 0.0037 by two-tailed Student’s *t*-test. The upper panel shows the compound binding motif of TAL1 and GATA1 next to an ETS binding motif, the gRNA target, and its protospacer adjacent motif (PAM) sequence. The predicted DNA cleavage site is shown (grey arrowhead). Note that a compound binding motif of TAL1 and GATA1 is a TG 7 or 8 bp upstream of a WGATAA motif and that Cas9 cleaves DNA 3 or 4 base pairs upstream from the PAM site. **c** Cas9 protein and control gRNA (upper) or gRNA targeting the *Myb* -68 enhancer (lower) were delivered by electroporation into oligopotent Ly6C^-^ GMPs. Representative FACS plots 4 (left) or 6 (right) days after electroporation are shown. FACS gating strategies are shown in Supplementary Fig. 14a. Numbers shown are mean percentages±SD of basophils, defined as FcεR1α^+^c-kit^-^ (blue), and mast cells, defined as FcεR1α^+^c-kit^+^ (red^)^. The percentages of basophils and mast cells are also shown in the right graph. *n* = 6 from 3 electroporation procedures. *p* = 0.0023, 0.00014, and 0.0023 from left to right by two-tailed Student’s *t*-test without adjustments for multiple comparisons.

To study whether the *Myb* -68 enhancer regulated *Myb* mRNA expression, we used clustered regularly interspaced short palindromic repeat (CRISPR)/CRISPR-associated protein 9 (Cas9) technologies. First we applied a CRISPR interference (CRISPRi) system, where a nuclease-inactive Cas9 fused to the KRAB repressor domain (dCas9-KRAB) reduces chromatin accessibility across a 1-2 kb window around a guide RNA (gRNA)’s target site^30,31^. As expected, dCas9-KRAB and doxycycline (DOX)-inducible gRNA targeting the *Myb* -68 enhancer suppressed *Myb* mRNA expression in mouse mast cell P815 cells but not in macrophage-like RAW 264.7 cells (Fig. 5a and Supplementary Fig. 4a). Next, to identify the core elements of the region, we used Cas9 to introduce a double-strand break at a compound binding motif of TAL1 and GATA1 next to an ETS binding motif. Specific disruption of the sequence was sufficient to partially decrease *Myb* mRNA expression only in mast cell P815 cells, suggesting the TAL1 and GATA1 motif was one of the key elements of the *Myb* -68 enhancer (Fig. 5b and Supplementary Fig. 4b). Further, to investigate the effect of Cas9-mediated perturbation of the *Myb* -68 enhancer on cellular differentiation, Cas9 protein and gRNA targeting the *Myb* -68 enhancer were delivered into oligopotent Ly6C^-^ GMPs by electroporation. Cas9-mediated destruction of the *Myb* -68 enhancer significantly decreased their ability to differentiate into basophils and mast cells (Fig. 5c), without affecting differentiation to other lineages (Supplementary Fig. 4c). Collectively, these findings indicate that the *Myb* -68 enhancer regulates *Myb* mRNA expression and basophil and mast cell differentiation.

### The *Myb* -68 enhancer-guided single cell RNA sequencing (scRNA-seq) shows the basophil differentiation trajectory in vivo

**Fig. 6:**
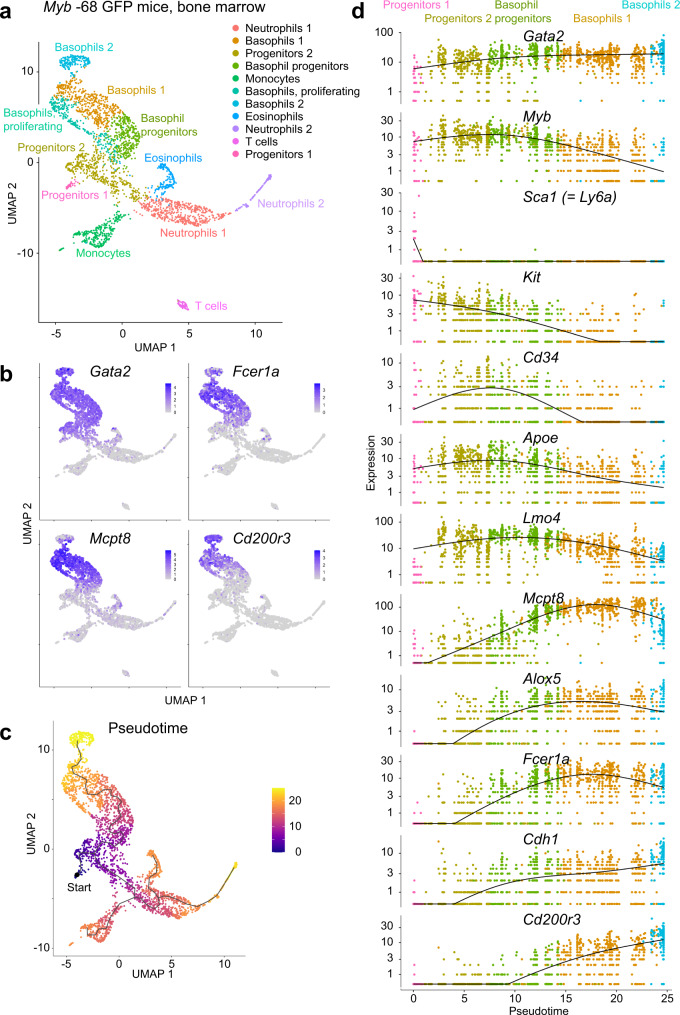
scRNA-seq analysis implicates an unperturbed in vivo differentiation trajectory of basophils. **a** Uniform manifold approximation and projection (UMAP) visualization of whole bone marrow GFP^+^ cells in *Myb* -68 GFP mice. Clusters were annotated according to the expression of known feature genes. Note that most neutrophil-lineage cells are *Mmp9*^-^ immature cells as shown in Supplementary Fig. 6a. **b** UMAP projection of selected basophil signature genes. **c** Trajectory analysis colored by pseudotime of each cell. Progenitors 1 were set as a defined starting point (black dot). **d** Pseudotime analysis for selected clusters: Progenitors 1 (pink), Progenitors 2 (dark yellow), Basophil progenitors (dark green), Basophils 1 (brown), and Basophils 2 (cyan). Expression levels of key lineage-associated genes are shown.

To elucidate detailed in vivo trajectories of basophil and mast cell differentiation in an unperturbed manner, scRNA-seq of whole bone marrow GFP^+^ cells in *Myb* -68 GFP mice was performed. Unsupervised clustering analysis identified 11 clusters, which were manually annotated according to the expression of known feature genes (Fig. 6a and Supplementary Figs. 5 and 6a). For example, the smallest cluster was “Progenitors 1” with HSC-related genes, *Ly6a* (= *Sca1*), *Procr*, and *Hlf*. Connected to Progenitors 1, the central cluster “Progenitors 2” showed features of FcεR1α^-^Ly6C^-^ GMPs with high expression of *Kit* and *Cd34* and no or low expression of *Sca1*, *Fcer1a*, *Ly6c2*, and other lineage markers.

Notably, four clusters were characterized with basophil markers including *Gata2*, *Fcer1a*, *Mcpt8*, and *Cd200r3* (Fig. 6b). In accord with previous reports^32^, *Kit* and *Cd34* downregulation was associated with upregulation of basophil specific genes (Supplementary Fig. 6a). Thus, using Progenitors 1 as a defined starting point, pseudotime analysis was performed (Fig. 6c), which confirmed a trajectory from Progenitors 1 toward Progenitors 2 and “Basophil progenitors”, and subsequently to “Basophils 1” and “Basophils 2” clusters. In contrast to previous in vitro experiments suggesting a close relationship between basophil and eosinophil differentiation^33^, our analysis demonstrated an independent basophil differentiation trajectory distinct from the other common trajectory diverging into eosinophil/monocyte/neutrophil-lineage cells.

While we were unable to detect any mature mast cells as shown by no mucosal (*Mcpt1* and *Mcpt2*), and no or very little connective tissue mast cell proteases (*Cma1*, *Tpsb2* and *Mcpt4*)^34^ (Supplementary Fig. 6b), the master regulator of mast cell differentiation, *Mitf*, was weakly expressed in a part of the Progenitors 2 cluster with *Itgb7*, a surface marker of previously defined bipotent basophil/mast cell progenitors (BMCP)^35–37^, *Cdh1* (= *E-cadherin*), another marker of pro-basophil and mast cell progenitors (pro-BMP)^38^, and *Il1rl1* (= *T1/ST2*), a marker of mast cell progenitors^36^, suggesting these cells might represent the bifurcation of basophil and mast cell differentiation (Supplementary Fig. 6c). HSC and megakaryocyte-related genes, *Mpl* and *Pf4*, were detected only in Progenitors 1 (Supplementary Fig. 6a), indicating that cell fate decision between basophils and megakaryocytes occurred much earlier than other lineage bifurcations.

Pseudotime analysis of individual genes implied early basophil differentiation markers including *Apoe*, *Lmo4*, *Csrp3*, *St8sia6*, *Casp3*, and *Tent5a*, and late markers including *Mcpt8*, *Alox5*, *Ms4a2* (= *Fcer1b*), *Fcer1a*, *Cdh1*, *Cpa3*, *Edem3*, *Ets1*, *Ptprs*, *Cd200r3*, and *Prss34* (= *Mcpt11*) (Fig. 6d and Supplementary Fig. 7). Early-onset genes with enzymatic activity included *St8sia6* (sialyltransferase to synthesize sialylglycoconjugates, thus producing ligands for Siglecs)^39^, *Casp3* (effector caspase in apoptosis), and *Tent5a* (nucleotidyltransferase responsible for mRNA polyadenylation)^40^, followed by *Alox5* (the key enzyme in the biosynthesis of leukotrienes) and *Cpa3* (carboxypeptidase involved in protein degradation)^41^, and finally well-known basophil tryptase *Mcpt8* and *Prss34*. The beta subunit of the high affinity IgE receptor *Ms4a2* preceded the alpha subunit *Fcer1a* and *Cdh1*, a pro-BMP marker, and they were followed by an activation marker *Cd200r3*. On the other hand, *Itgb7* and *Il1rl1* showed no significant increase in basophil progenitors (Supplementary Fig. 7e).

### LILRB4 is a maturation marker of basophils

**Fig. 7:**
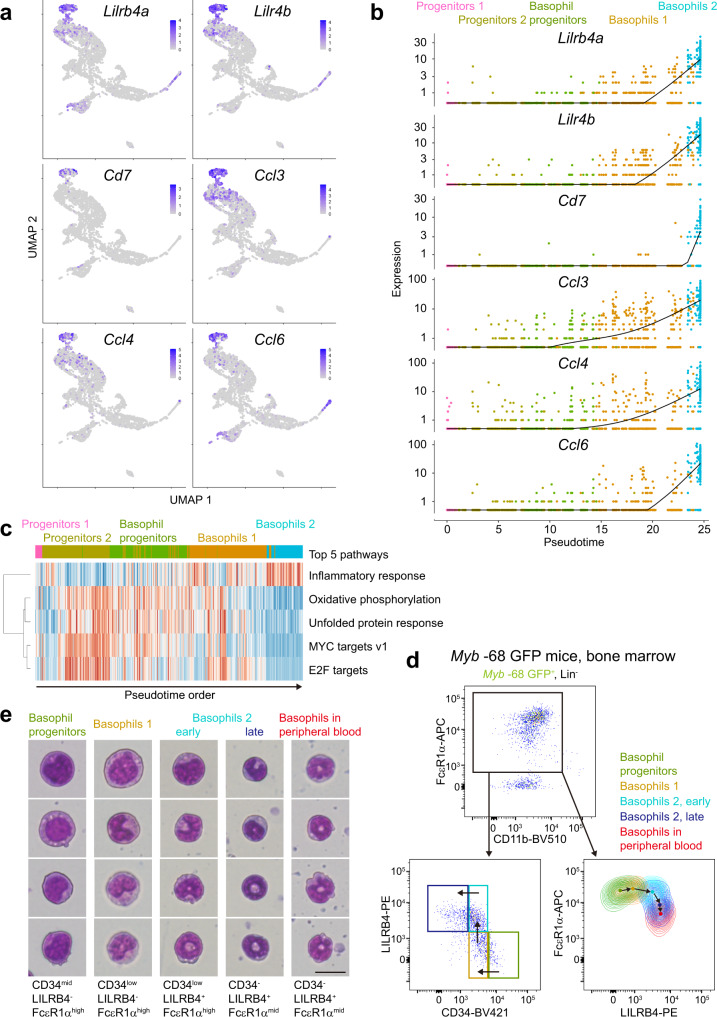
High-resolution fractionation of immature and mature basophils by scRNA-seq and FACS analysis. **a** UMAP projection of selected Basophils 2 hallmark genes. **b** Pseudotime analysis for selected clusters: Progenitors 1 (pink), Progenitors 2 (dark yellow), Basophil progenitors (dark green), Basophils 1 (brown), and Basophils 2 (cyan). Expression levels of selected Basophils 2 signature genes are shown. **c** Gene set variation analysis comparing Basophils 1 and Basophils 2. The top 5 differentially enriched pathways were shown. **d** FACS plots of bone marrow cells from *Myb* -68 GFP mice. FACS gating strategies are shown in Supplementary Fig.12. The top panel shows a plot of GFP^+^Lin^-^ cells. Lin^-^ indicates CD4^-^CD8^-^B220^-^Gr1^-^Ly6C^-^CD11c^-^cKit^-^Ter119^-^SiglecF^-^IL7Ra^-^NK1.1^-^. The left lower panel shows that GFP^+^Lin^-^FcεR1α^+^ cells are subdivided into 4 groups: Basophil progenitors (CD34^mid^LILRB4^-^FcεR1α^high^, dark green), Basophils 1 (CD34^low^LILRB4^-^FcεR1α^high^, brown), early Basophils 2 (CD34^low^LILRB4^+^FcεR1α^high^, cyan), and late Basophils 2 (CD34^-^LILRB4^+^FcεR1α^mid^, blue). The right lower panel is a FACS contour plot showing LILRB4 and FcεR1α expression levels of each subset defined in the left lower panel. In addition, data obtained from basophils in peripheral blood (red) were overlaid for comparison. Small black arrows inside plots show the direction of basophil differentiation. Representative plots from 3 independent experiments are shown. **e** May Grünwald Giemsa staining of each subset of basophil-lineage cells. Bar shows 20 μm.

Next we focused on the difference between Basophils 1 and Basophils 2. Genes elevated in Basophils 2 included *Lilrb4a* and *Lilr4b* (orthologs for human *LILRB4*), *Cd7*, and inflammatory CC chemokines (*Ccl3*, *Ccl4*, *Ccl6*, and *Ccl9*) (Figs. 7a, b and Supplementary Fig. 8). The difference of mRNA expression levels between Basophils 1 and Basophils 2 was larger with *Lilrb4a*, *Lilr4b* and *Cd7* than with other conventional basophil surface markers, *Itga2* (= *Cd49b*) and *Cd200r3* (Supplementary Fig. 8a). Gene set variation analysis showed that genes related to oxidative phosphorylation and unfolded protein response were downregulated and genes related to inflammatory response were upregulated in Basophils 2 (Fig. 7c). *Sytl3*, a critical regulator of terminal transport and secretion of granules^42^, was also upregulated in Basophils 2 (Supplementary Fig. 8b, c).

To fortify and refine the differentiation trajectory identified by single-cell transcriptomics data, we analyzed Lin^-^FcεR1α^+^ basophil-lineage cells in bone marrow by FACS using anti-LILRB4 antibodies (Fig. 7d). Cells were subdivided into 4 groups: Basophil progenitors (CD34^mid^LILRB4^-^FcεR1α^high^), Basophils 1 (CD34^low^LILRB4^-^FcεR1α^high^), early Basophils 2 (CD34^low^LILRB4^+^FcεR1α^high^), and late Basophils 2 (CD34^-^LILRB4^+^FcεR1α^mid^). CD34 downregulation was coupled with LILRB4 elevation, and FcεR1α expression peaked around the Basophils 1 stage, and then decreased with LILRB4 induction. The majority of peripheral blood basophils expressed LILRB4, further reinforced the notion that Basophils 2 were the most mature basophils in bone marrow.

We sorted these cells to determine their morphological identity (Fig. 7e). As expected, Basophils 1 were relatively large, progenitor-like cells with round or slightly indented nuclei. Basophils 2 were smaller cells with C-shaped or ring-like nuclei and less cytoplasm, and at the later stage they were almost indistinguishable from basophils in peripheral blood. These findings are in accord with our scRNA-seq data, and suggest that LILRB4^+^ Basophils 2 are the most differentiated basophils in bone marrow.

### Integration of two scRNA-seq datasets identified bone marrow cells with a gene signature of immature mast cells

**Fig. 8:**
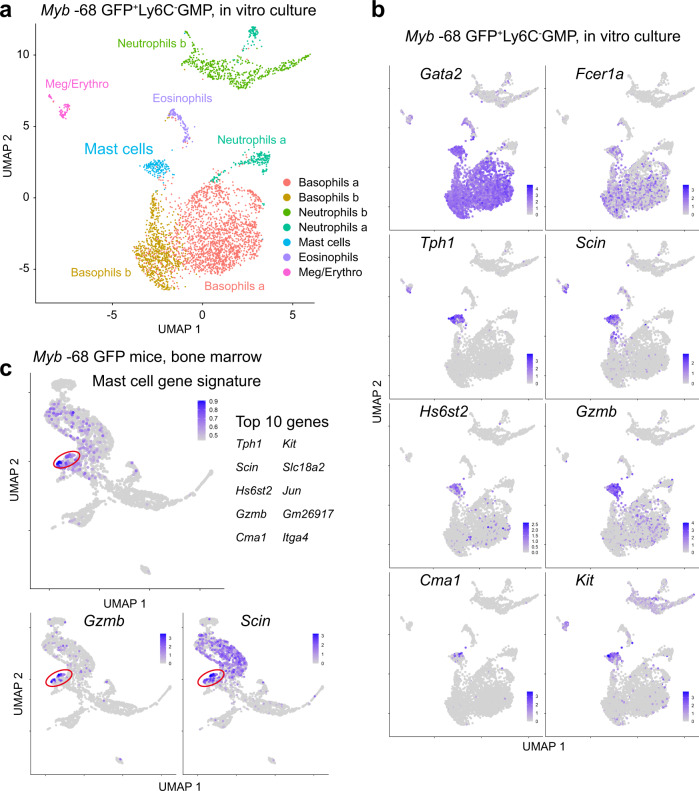
Integration of two scRNA-seq datasets identified bone marrow cells with a gene signature of immature mast cells. **a** UMAP visualization of in vitro mast cell culture. FACS-sorted *Myb* -68 GFP^+^ FcεR1α^-^Ly6C^-^ GMPs were cultured with IL-3 for 3 days, and then analyzed. Clusters were annotated according to the expression of known feature genes. **b** UMAP projection of selected mast cell signature genes. **c** UMAP projection of the mast cell gene signature (upper) and selected mast cell genes (lower). The top 10 marker genes to characterize immature mast cells (upper right) were determined using scRNA-seq data of in vitro mast cell culture, and enrichment of the gene signature in bone marrow GFP^+^ cells in *Myb* -68 GFP mice was calculated by gene set variation analysis. Cells with enrichment of the mast cell gene signature were shown in red. Cells were displayed in order of expression to prevent cells with high expression from getting buried.

Because our scRNA-seq analysis of bone marrow cells were unable to detect mast cell-biased cells clearly, we conducted the second scRNA-seq using in vitro mast cell culture, aiming to identify a gene signature of immature mast cells (Fig. 8 and Supplementary Figures 9 and 10). *Myb* -68 GFP^+^ FcεR1α^-^Ly6C^-^ GMPs were cultured with IL-3 for 3 days, and then analyzed by scRNA-seq. Similarly with a previous report showing that FcεR1α^+^ GMPs still retained the differentiation capacity to non-basophil/mast cell lineages^43^, some GFP^+^ cells gave rise to other lineages. However, most cells differentiated into basophil-lineage cells with *Gata2*, *Fcer1a*, *Mcpt8*, *Prss34*, *Lilrb4a* and *Lilr4b* but without *Cd34* and *Kit*, and a small portion of cells showed characteristics of mast cells with *Gata2*, *Fcer1a*, *Kit* and *Cma1* (= *Mcpt5*) expression (Figs. 8,b and Supplementary Fig. 10a). Differentially expressed gene analysis determined the top 10 marker genes to characterize the mast cell cluster: *Tph1* (the rate-limiting enzyme in the biosynthesis of serotonin), *Scin* (*Scinderin*, actin filament-severing protein to regulate exocytosis)^44^, *Hs6st2* (an enzyme to synthesize heparan sulfate necessary for the storage of mast cell proteases)^45^, *Gzmb* (*Granzyme b*)^46^, *Cma1* (*Chymase 1*), *Kit*, *Slc18a2* (a transporter of monoamines such as serotonin and histamine), *Jun*, *Gm26917*, and *Itga4* (a binding partner of Integrin β7) (Fig. 8b and Supplementary Fig. 10b).

Next we re-analyzed the first scRNA-seq data of bone marrow cells to find cells with features of immature mast cells. Although the top marker gene *Tph1* was not detected in bone marrow, gene set variation analysis demonstrated that a subset of Progenitors 2, located in the same area with weak *Mitf* expression (Supplementary Fig. 6c), showed enrichment of the mast cell gene signature with high expression of *Gzmb* and *Scin* (Fig. 8c and Supplementary Fig. 10c). Interestingly, these cells and erythrocyte-primed cells with *Car1* and *Mfsd2b* expression were positioned close to each other, in line with recent reports showing coupling between the erythroid and the basophil or mast cell fates^47^.

While IL-3 is well known to promote basophil and mast cell differentiation, thymic stromal lymphopoietin (TSLP) also enhances their differentiation in an IL-3-independent manner. Previously published microarray data demonstrated that *Gzmb* and *Scin* were highly expressed also in TSLP-elicited Lin^-^CD34^+^cKit^+^ progenitors^48^. In addition, other TSLP-induced genes, including *Clnk* (= *Mist*), a regulator of mast cell degranulation^49^, were also co-expressed with *Gzmb* and *Scin* in our scRNA-seq data (Supplementary Fig. 10d).

To further corroborate the existence of cells primed toward mast cell differentiation, we integrated the two scRNA-seq datasets into one differentiation map (Supplementary Fig. 11), which clearly showed the overlap of a subset of Progenitors 2 in bone marrow and mast cells cultured in vitro, with high *Gzmb* and *Scin* expression in both. Taken together, these data have confirmed that bone marrow cells with a mast cell gene signature are characterized by high expression of *Gzmb* and *Scin*, and they are better markers than mucosal or connective tissue mast cell proteases.

### Ly6C^-^ GMPs with very high expression of Integrin β7 have intracellular Granzyme b and are biased to mast cell differentiation, but not to basophil differentiation

**Fig. 9:**
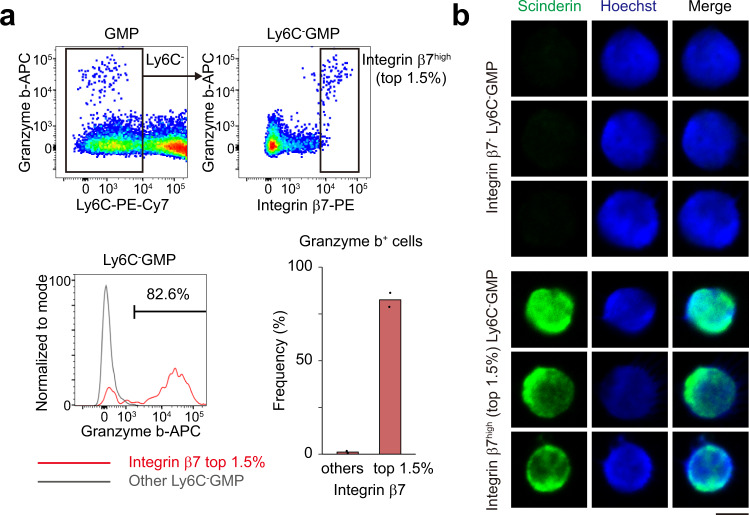
Ly6C^-^ GMPs with very high expression of Integrin β7 have intracellular Granzyme b and Scinderin. **a** Granzyme b expression in GMPs (left) and Ly6C^-^ GMPs (right) analyzed by FACS. FACS gating strategies are shown in Supplementary Fig. 14b. The left lower histogram compares the expression of Granzyme b between Integrin β7^high^ Ly6C^-^ GMPs (red, those ranked in the top 1.5% of all Ly6C^-^ GMPs) and other Ly6C^-^ GMPs (grey). The percentages of Granzyme b^+^ cells are also shown in the right lower graph. *n* = 2 mice. **b** Integrin β7^-^ (upper) and Integrin β7^high^ (top 1.5%, lower) Ly6C^-^ GMPs were stained with anti-Scinderin antibody (green) and Hoechst (blue). FACS gating strategies are shown in Supplementary Fig.12. Three representative cells for each from 2 independent experiments are shown. Bar shows 5 μm.

**Fig. 10:**
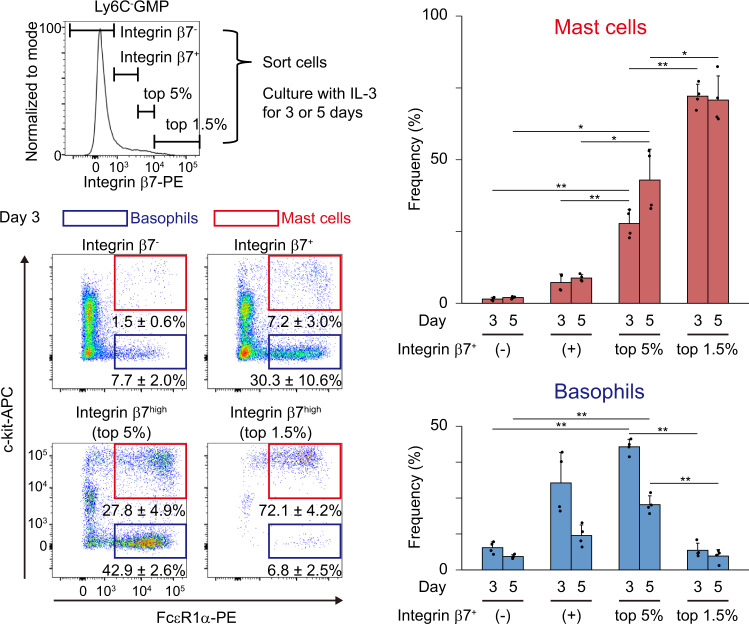
Ly6C^-^ GMPs with very high expression of Integrin β7 are biased to mast cell differentiation. Ly6C^-^ GMPs were sorted and divided into 4 groups: Integrin β7^-^ cells, Integrin β7^+^ cells (those did not rank in the top 5% of all Ly6C^-^ GMPs), cells with high expression of Integrin β7 (those ranked between the top 1.5% and 5%), and cells with very high expression of Integrin β7 (those ranked in the top 1.5%). FACS gating strategies for sorting are shown in Supplementary Fig. 12. Cells were cultured in vitro in the presence of IL-3 for indicated periods of time, and then analyzed. FACS gating strategies for analysis are shown in Supplementary Fig. 14a. Numbers shown are mean percentages±SD of basophils, defined as FcεR1α^+^c-kit^-^ (blue), and mast cells, defined as FcεR1α^+^c-kit^+^ (red). The percentages of basophils and mast cells are also shown in the right graph. *n* = 4 from 2 mice and from 2 independent experiments. *p* = 2.4 × 10^−^^6^, 2.8 × 10^−^^5^, 2.4 × 10^−^^6^, and 4.2 × 10^−^^4^ for mast cells on day 3, mast cells on day 5, basophils on day 3, and basophils on day 5, respectively, by one-way Welch’s ANOVA. ^*^p < 0.05 and ^**^p < 0.01 by the Games-Howell post hoc test. For mast cells on day 3, *p* = 0.0047, 0.0032, and 4.5 × 10^−^^5^ from left to right. For mast cells on day 5, *p* = 0.014, 0.023, and 0.028 from left to right. For basophils on day 3, *p* = 5.2 × 10^−^^6^ and 4.4 × 10^−^^6^ from left to right. For basophils on day 5, *p* = 0.0029 and 5.2 × 10^−^^4^ from left to right.

Because our scRNA-seq analysis indicated that *Gzmb* and *Scin* were markers of mast cell differentiation, next we examined their protein expression in GMPs. While most GMPs had no Granzyme b, it was induced predominantly in Ly6C^-^ GMPs with very high expression of Integrin β7 (those ranked in the top 1.5% of all Ly6C^-^ GMPs) (Fig. 9a). Immunocytochemistry indicated that Integrin β7^high^ cells also had cytoplasmic Scinderin (Fig. 9b). Thus, we used Integrin β7 to sort cells with mast cell differentiation potential prospectively (Fig. 10). In line with previous reports that showed Integrin β7 is a marker of BMCPs^35–37^, Integrin β7 expression among Ly6C^-^ GMPs was generally correlated with the capacity to differentiate into both mast cells and basophils. Ly6C^-^ GMPs with relatively high expression of Integrin β7 (those ranked between the top 1.5% and 5%) exhibited high balanced potential toward mast cells and basophils. Intriguingly, in contrast, Ly6C^-^ GMPs with very high expression of Integrin β7 (the top 1.5%) gave rise to significantly less basophils than other subsets of Integrin β7^+^ cells, and showed the highest potential to mast cell differentiation, indicating that most of these cells were mast cell progenitors losing the ability to differentiate to basophils. Collectively, these in vitro data have confirmed that Ly6C^-^ GMPs with very high expression of Integrin β7 have intracellular Granzyme b and Scinderin, and are primed to mast cell differentiation, but not to basophil differentiation, supporting the findings of our scRNA-seq analysis.

## Discussion

By analyzing multiple sets of ChIP-seq data, we selected several candidate cis-regulatory elements of the key hematopoietic regulator gene *Myb*. We exploited zebrafish as an in vivo validation model, and identified 2 enhancer regions active in zebrafish hematopoietic organs (Fig. 1). Then we generated transgenic mice with an EGFP reporter construct linked to these elements and confirmed their specificity in mouse hematopoiesis.

In *Myb* -74 GFP mice, Pre-Pro-B cells and ETPs were marked by EGFP, and the intensity gradually declined along B cell and T cell maturation (Supplementary Fig. 2). This element was selected by ChIP-seq of human T-ALL Jurkat cells which showed the binding of TAL1, TCF3 and TCF12 to this region (Fig. 1b). Because this region contains multiple TAL1, TCF3 and TCF12 binding motifs (E-box, CANNTG), it is reasonable to speculate that these transcription factors are the main regulators of the *Myb* -74 enhancer activity. Whereas TCF3 and TCF12 are consistently expressed along T cell and B cell development, TAL1 expression is normally silenced during their maturation, which may explain the attenuation of the *Myb* -74 enhancer activity in normal lymphopoiesis.

Detailed analysis of *Myb* -68 GFP mice showed that the *Myb* -68 enhancer activity was a feature of the basophil/mast cell lineage (Figs. 2 and 3), and able to enrich basophil/mast cell-biased GMPs (Fig. 4). ChIP-seq data from primary mast cells^29^ demonstrate that the key mast cell regulator MITF binds to the *Myb* -68 enhancer, which can explain strong and persistent activity of the *Myb* -68 enhancer in mast cells. The absence of MITF in basophils may be responsible for gradual loss of the activity in basophils. While disruption of the TAL1/GATA1 motif reduced *Myb* mRNA expression (Fig. 5b), we speculate that the binding motif of MITF is another key element of the *Myb* -68 enhancer. The absence of protospacer adjacent motif (PAM) sequences nearby hindered the experimental disruption of the MITF motif by CRISPR/Cas9. In addition, the CRISPR/Cas9 and dCas9-KRAB systems targeted for enhancers can suffer from insufficient knock-down and poor performance. Potential technical caveats include (1) highly variable efficiency among different sgRNA, (2) local chromatin effects, (3) competition between Cas9 and endogenous transcriptional regulators, and (4) interference from already present epigenetic marks. Another potential biological cause for incomplete suppression is the existence of shadow enhancers, a safeguard mechanism against genetic deletion of one enhancer^31,50–52^. Genes with critical functions are commonly regulated by multiple enhancers to mask the effects of perturbing individual enhancers and confer robustness to gene regulation. The *Myb* -68 enhancer also can have shadow enhancers. Further studies are necessary to elucidate the molecular mechanism that regulates the *Myb* -68 activities and *Myb* mRNA expression in more detail.

Basophils are the rarest granulocytes representing less than 1% of peripheral blood leukocytes. Recent studies have discovered that they regulate not only Th2 responses in allergy and IL4 secretion after helminth infection^53^, but also innate immunity^54^, tissue development^55^, and tissue destruction^56^. However, due to their rarity and no definite surface markers, it has been challenging to define the in vivo differentiation journey of basophils comprehensively from HSPCs to mature basophils. Recently, scRNA-seq of mouse bone marrow Lin^-^ckit^+^ progenitors demonstrated a differentiation trajectory from HSCs to BMCPs^37^. scRNA-seq comparing CD34^+^ basophil progenitors and CD34^-^ basophils was also reported^32^. Here we defined their differentiation trajectory continuous from HSPCs to differentiated basophils during normal hematopoiesis (Figs. 6 and 7). GFP activity of lineage-committed GPs and MPs in *Myb* -68 GFP mice are less than 1%. Nonetheless, due to their overwhelming abundance, a non-negligible number of non-basophil lineage cells were also detected in our scRNA-seq. The modest selectivity of *Myb* -68 GFP mice serendipitously enabled us to identify transcriptome difference among basophil and other lineages in one analysis. Although our study is unable to deny the possible existence of alternative *Myb* -68 independent differentiation pathways, our scRNA-seq data, together with previously published data, will provide us a reference map of transcriptional changes in basophil differentiation in bone marrow.

*Ccl3* expression in Basophils 2 was in line with previous findings that basophils in bone marrow constitutively expressed CCL3 without any stimuli^57^. On the other hand, surface expression of LILRB4 and CD7 on basophils was not previously appreciated. Accordingly, we highlighted LILRB4 as a marker of mature basophils (Fig. 7). LILRB4 is a member of the leukocyte immunoglobulin-like receptor (LILR) family, and mainly expressed on antigen-presenting cells including dendritic cells and macrophages^58,59^. Its cytoplasmic region has four immunoreceptor tyrosine-based inhibitory motifs (ITIM), which can recruit the phosphatases SHP-1, SHP-2, and SHIP, and inhibit immune cell activation. Historically, studies on immune inhibitory receptors have been focused on NK cells, macrophages, dendritic cells, neutrophils, eosinophils, and mast cells^59^. The function of immune inhibitory receptors on basophils is largely unknown. In addition, *Ptprs*, an inhibitory receptor-type protein tyrosine phosphatase previously thought to be specific to plasmacytoid dendritic cells (pDC)^60^, was also broadly expressed in immature basophil-lineage cells, further discovering unexpected similarity between basophils and pDCs. Further research is likely to disclose hitherto unknown immunological properties of basophils.

Finally, by projecting scRNA-seq data of mast cell culture on in vivo differentiation trajectories, we identified bone marrow cells primed toward mast cell differentiation (Fig. 8). Our analysis implies that mast cell markers including *Mcpt1*, *Mcpt2*, *Tpsb2*, *Mcpt4* and *Tpsg1* are relatively useless to characterize the mast cell lineage in bone marrow, and underscores the importance to use a gene signature of immature mast cells, especially *Gzmb* and *Scin* expression, in accord with previous reports^37,47^. While *Gzmb* was established as one of the earliest proteases to characterize mast cell-lineage cells, our scRNA-seq data were unable to determine their definite surface markers. Because Ly6C^-^ GMPs with very high expression of Integrin β7 were shown to be biased to mast cell differentiation, but not to basophil differentiation (Figs. 9 and 10), very high expression of Integrin β7, or a combination of *Itgb7* and *Itga4* (Integrin α4β7 or LPAM-1), can be a useful marker to prospectively enrich mast cell-lineage cells in bone marrow.

Collectively, our study shows how a combination of lineage-associated reporter mice and single-cell transcriptomics can overcome the rarity of target cells and enhance our understanding of gene expression programs that control cell differentiation in vivo. Our *Myb* -68 GFP mice will pave the way for various in vivo analyses previously difficult to conduct due to a paucity of basophils and mast cells.

## Methods

### Animals

All animal procedures were approved by the Institutional Animal Care and Use Committee of the National University of Singapore and were performed according to their recommendations. For zebrafish microinjection, the EGFP reporter constructs were generated by subcloning genomic fragments of mouse candidate enhancer regions into the upstream of the zebrafish heat shock protein 70 minimal promoter (zhsp70p) and EGFP of the ISceI-pBSII SK + vector^17^. The plasmids were resuspended in buffer and linearized using the I-SceI enzyme (New England Biolabs, R0694S). The wild-type zebrafish (Danio rerio) AB line was maintained, and the linearized plasmid solution was injected into one-cell-stage zebrafish embryos. Zebrafish embryo sex was not examined. Each construct was injected more than 100 eggs in one experiment, and at least two independent experiments were conducted for each construct. For generation and maintenance of transgenic mice, the EGFP reporter constructs were made by subcloning genomic fragments of either the *Myb* -74 kb or -68 kb enhancer region into the upstream of the mouse heat shock protein 68 minimal promoter (mhsp68p) and EGFP of the ISceI-pBSII SK + vector, and *Myb* -74 kb and -68 kb enhancer element-EGFP transgenic mice were generated^17^. All mice were maintained on a C57BL/6 background under specific pathogen-free conditions in a 12/12 h light/dark cycle with food and water provided ad libitum. The room temperature for mice was between 20 °C and 26 °C, and the relative humidity was kept at between 30% and 70%. Mice were assigned to experimental groups based on their genotypes, and 8 to 16 weeks old, littermate-, age- and gender-matched mice were used. Animal care was in accordance with the guidelines of National University of Singapore. *Myb* -74 kb and -68 kb enhancer element-EGFP transgenic mice are available from the authors upon reasonable request.

### Analysis of previously published ChIP-seq and Hi-ChIP data

Previously published ChIP-seq data (GSE22178, GSE29181, and ERA000161)^19–21^ were downloaded, and reads that passed the quality filter step were mapped to the reference mouse or human genome sequence (mm10 or hg38) using Bowtie2^61^. Coverage tracks were generated by deepTools^62^, and visualized by the Integrative Genomics Viewer^63^. For GSE59636^8^ and GSE48086^29^, bigwig and bedgraph files deposited in GEO were visualized by the Integrative Genomics Viewer. Mouse T-ALL cells Hi-ChIP data^23^ was analyzed by Hi-C Pro^64^ and hichipper^65^.

### FACS analysis and sorting of mouse bone marrow cells and peripheral blood cells

Bone marrow cells were harvested from femurs, tibias, and the spine of 2- to 4-month-old age- and sex-matched mice. The cells were dissociated to a single-cell suspension by filtering through a 70 μm nylon mesh. To analyze peripheral blood cells, 1.2% dextran in PBS was added to blood to sediment erythrocytes for 45 min at room temperature, and then the leukocyte-rich plasma above the sedimented erythrocytes was used. Cells were Fc-blocked and stained with anti-mouse primary antibodies for 60 min. All antibodies were purchased from Thermo Fisher, BD Biosciences or BioLegend. For sorting, cKit^+^ cells were pre-enriched with the CD117 MicroBeads and the MACS LS columns (Miltenyi Biotec, 130-091-224 and 130-042-401). For intracellular staining, cells were stained using Cytofix/Cytoperm Fixation/Permeabilization Solution (BD Biosciences, 555028). Cells were analyzed or sorted using LSRII and FACS Aria II cytometers (BD Biosciences). Subsequent data analyses were performed with the FlowJo analysis software (FlowJo, LLC). For morphological features, cytospin preparations were stained with May-Grünwald solution and Giemsa Stain, Modified Solution (Sigma).

### FACS antibodies

The following antibodies were used for flow cytometric analysis. B220 (RA3-6B2) (Biolegend, 103207/103223/103235/103239/103247, BD, 553092/552772), CD3e (145-2C11) (Biolegend, 100327), CD4 (RM4.5/GK1.5) (Biolegend, 100413/100540, eBioscience, 17-0041-81), CD8a (53-6.7) (Biolegend, 100714/100734, BD, 552877), CD11b (M1/70) (Biolegend, 101228/101235/101245), CD11c (N418) (Biolegend 117323/117327), CD16/CD32 (93) (Biolegend, 101333), CD24 (M1/69) (Biolegend, 101807/101814), CD25 (PC61) (Biolegend, 102012), CD34 (RAM34) (eBioscience, 50-0341-82), CD34 (SA376A4) (Biolegend, 152203/152207), CD41 (MWReg30) (BD, 558040), CD44 (IM7) (BD, 553134), CD49b (DX5) (Biolegend, 108919/10892, eBioscience, 17-5971-81), CD62L (MEL-14) (Biolegend, 104411), CD105 (MJ7/18) (Biolegend, 120409), CD115 (AFS98) (BD, 566839), CD135 (A2F10) (Biolegend, 135305/135313, eBioscience, 17-1351-82), CD117 ( = c-kit) (2B8) (Biolegend, 105811/105823/105826/105827, BD, 558163), CD135 (A2F10) (Biolegend, 135305, eBioscience, 17-1351-82), CD150 (TC15-12F12.2) (Biolegend, 115904/115909/115914), F4/80 (BM8) (Biolegend 123113/123115/123132), FcεR1α (MAR-1) (Biolegend 134307/134315/134318), Gr1 (RB6-8C5) (Biolegend, 108408/108428/108412), Granzyme b (QA16A02) (Biolegend, 372203), IgD (11-26 c.2a) (Biolegend, 405713), IgM (RMM-1) (Biolegend, 406507), IL7Rα (A7R34) (eBioscience, 12-1271-82, Biolegend, 135021), LILRB4 (H1.1) (Biolegend, 144904), Ly6C (HK1.4) (Biolegend, 128011/128015/128017), Ly6G (1A8) (Biolegend, 127607/127613), Ly51 (6C3) (Biolegend, 108307/108313, eBioscience, 17-5891-80), NK1.1 (PK136) (Biolegend, 108727), Sca1 (D7) (eBioscience, 17-5981-83, Biolegend, 108114/108126/108129), Siglec-F (E50-2440) (BD, 552126/565526), and Ter119 (TER-119) (Biolegend, 116228). All antibodies are validated by each manufacturer to detect mouse cells by FACS. All antibodies were used at 1:100 dilution.

### Quantitative reverse transcription (RT)-PCR assay

Total RNA was extracted and reverse transcribed using the Taqman Fast Cells-to-CT Kit (Thermo Fisher, 4399003). Real-time quantitative RT-PCR was performed using the Taqman PreAmp Master Mix (Thermo Fisher, 4391128), Taqman Fast Universal PCR Master Mix (Thermo Fisher, 4366072), and a QuantStudio 3 Real-Time PCR System (Thermo Fisher). In some experiments, total RNA was extracted using the RNeasy Micro Kit (Qiagen, 74004) and reverse transcribed using the EvoScript Reverse Transcriptase (Roche, 07912315001). Real-time quantitative RT-PCR was performed using the TB Green Premix Ex Taq II (Tli RNaseH Plus) (Takara, RR820A) and a Takara Thermal Cycler Dice Realtime System (Takara). Relative expression was calculated for each gene by the comparative CT method and with *Actb* for normalization. Probes were shown in Supplementary Fig. 2.

### In vitro cell culture

Sorted bone marrow cells were cultured in StemSpan SFEM medium (STEMCELL Technologies) supplemented with mouse recombinant GM-CSF (20 ng/ml, Peprotech) or mouse recombinant IL-3 (20 ng/ml, Peprotech). Mouse mast cell P815 cells were provided by the RIKEN BRC through the National BioResource Project of the MEXT, Japan, and maintained in RPMI-1640 (Wako) supplemented with 10% fetal bovine serum and Antibiotic-Antimycotic (Thermo Fisher). RAW264.7 cells were maintained in D-MEM (High Glucose, Wako) supplemented with 10% fetal bovine serum and Antibiotic-Antimycotic (Thermo Fisher).

### Lentiviral infection

FUCas9Cherry, a lentiviral expression vector for Cas9 with an mCherry marker, and FgH1tUTG, a lentiviral vector for DOX-inducible gRNA with a GFP marker, were gifts from Marco Herold (Addgene plasmid # 70182 and #70183)^66^. The gRNA sequence targeting the *Myb* -68 element (CCTTCTGTACTGTCAAGATA) was cloned into FgH1tUTG. pLV-UBC-3xFLAG-dCas9-KRAB-T2A-mCherry, a lentiviral expression vector for dCas9-KRAB with an mCherry marker, was designed and purchased from VectorBuilder. Lentiviral supernatant was generated by transient transfection of HEK293T cells using FuGENE HD (Promega, E2312) or PEI MAX (Polysciences, 24765-100) according to the manufacturer’s instructions, and concentrated with Amicon Ultra-15 Centrifugal Filter Unit (Millipore, UFC910024) when necessary. Cells were spin-infected at 1300 x g, or infected using ViroMag (OZ Biosciences, VM41000) according to the manufacturer’s instructions. GFP^+^ and mCherry^+^ cells were selected by flow cytometry. For DOX-inducible gRNA expression, doxycycline hyclate (Sigma) was used. The T7 endonuclease I (T7EI) mismatch detection assay was conducted using T7 Endonuclease I (New England Biolabs, M0302L). The PCR primer sequences used for the assay are 5**′**-ACTATGCCACACAGTCTGCA-3**′** and 5**′**-CCACTAGAGACAGAAACTGC-3**′**.

### Electroporation

Following pre-culture for 1 day, sorted Ly6C^-^ GMPs were electroporated using Alt-R CRISPR-Cas9 crRNA targeting the *Myb* -68 element (CCTTCTGTACTGTCAAGATA), Alt-R CRISPR-Cas9 tracrRNA, ATTO 550, Alt-R S.p. Cas9 Nuclease V3, Alt-R Cas9 Electroporation Enhancer (Integrated DNA Technologies, 1075928, 1081058, and 1075916), and the Neon Transfection System (1700 V, 20 ms, 1 pulse) (Thermo Fisher) according to the manufacturer’s instructions. Cells were cultured in StemSpan SFEM medium (STEMCELL Technologies) supplemented with mouse recombinant IL-3 (20 ng/ml, Peprotech) for 24 h and then ATTO 550^+^ cells were sorted for further analysis.

### Immunocytochemistry

Cells were fixed and permeabilized using ice-cold methanol and 0.1% Tris-buffered saline with 0.1% Tween 20, blocked, and then stained with rabbit polyclonal anti-SCIN antibody (Novus, NBP1-31721, 1:250, validated by Novus). Fluorescent images were collected with a confocal laser microscope (LSM880, Zeiss).

### scRNA-seq analysis

scRNA-seq was performed on the 10X Genomics platform using the Chromium Controller and the Chromium Next GEM Single Cell 3ʹ Kit v3.1 (10x Genomics, 1000268) following the manufacturer’s instructions. To remove macrophages engulfing GFP, GFP^+^F4/80^-^ cells were sorted twice from whole bone marrow cells of *Myb* -68 GFP mice. For in vitro analysis, FACS-sorted *Myb* -68 GFP^+^ FcεR1α^-^Ly6C^-^ GMPs were cultured with IL-3 (20 ng/ml) for 3 days, and then harvested. Cells were loaded aiming for a targeted cell recovery of 2,000 cells. The quality of the obtained cDNA library was assessed by Bioanalyzer (Agilent). Sequencing was performed on a DNBSEQ sequencer (BGI). Raw unique molecular identifier (UMI)-based data files were mapped against the mm10 reference genome, and mapped reads were counted using the Cell Ranger package (10x Genomics) with default parameters. The Seurat package^67^ in R was used to analyze the scRNA-seq data. Clusters were detected using FindClusters, and annotated based on feature genes. To mitigate the effects of cell cycle heterogeneity, cell-cycle scoring and regression were performed using CellCycleScoring and ScaleData. Differentially expressed genes were identified by running FindAllMarkers. The Monocle3 package^68^ in R was used to determine the pseudotime of basophil differentiation. Cells belonging to basophil lineage, namely Progenitors 1, Progenitors 2, Basophil progenitors, Basophils 1, and Basophils 2, were selected and used to construct single-cell trajectories. Gene set variation analysis (GSVA) was conducted by using the GSVA package^69^ in R and hallmark gene sets from the Molecular Signatures Database (MSigDB). Differentially enriched gene sets between Basophils 1 and Basophils 2 were determined and ranked by the limma package^70^ in R. Two scRNA-seq datasets were integrated by running FindIntegrationAnchors and IntegrateData in the Seurat package.

### Statistical analysis

All values were presented as means ± SD. Except for scRNA-seq, statistical analysis was performed using two-tailed Student’s *t*-test, one-way analysis of variance (ANOVA) with the Tukey-Kramer post hoc test, or Welch’s ANOVA with the Games-Howell post hoc test, and values were considered to be significant at *p* < 0.05.

### Reporting summary

Further information on research design is available in the Nature Portfolio Reporting Summary linked to this article.

## Supplementary information

Supplementary Information

Reporting Summary

## Data Availability

The scRNA-seq data generated in this study have been deposited in the Gene Expression Omnibus database: accession numbers GSE207688 and GSE207689. Previously published sequencing data (GSE22178, GSE29181, ERA000161, GSE59636, GSE48086, and GSE115363) were available from each site. Reference mouse and human genome sequences (mm10 and hg38) are available from iGenomes (Illumina, https://support.illumina.com/sequencing/sequencing_software/igenome.html). Source data are provided with this paper. The data that support this study are available from the corresponding authors upon request. Source data are provided with this paper.

## Source data

Source Data
