# Supplementary material for: A Myb enhancer-guided analysis of basophil and mast cell differentiation
Source: Nat Commun. 2022 Nov 18;13:7064. doi: 10.1038/s41467-022-34906-1 (PMC9674656; doi:10.1038/s41467-022-34906-1)
Supplement: Supplementary file 1 — Supplementary Information [file 41467_2022_34906_MOESM1_ESM.pdf]

SUPPLEMENTARY INFORMATION

A *Myb* enhancer-guided analysis of basophil and mast cell differentiation

Takayoshi Matsumura, et al.

SUPPLEMENTARY TABLES 1 and 2 pages 2 and 3

SUPPLEMENTARY FIGURES 1 to 14 pages 4 to 31

SUPPLEMENTARY TABLE

Supplementary Table 1. Candidate enhancer regions around the *Myb* locus selected by in silico analysis.

Name	Position (mm10)	around RIS clusters	ChIP-seq peaks		
			HPC-7	EML	Jurkat*
#1: -91 kb	chr10: 21252216-21252519	+	-	-	+
#2: -81 kb	chr10: 21241938-21242148	+	-	+	-
#3: -74 kb	chr10: 21235083-21235566	+	-	-	+
#4: -68 kb	chr10: 21228355-21228914	+	+	+	-
#5: -36 kb	chr10: 21196483-21197033	-	+	+	-
#6: +14 kb	chr10: 21146267-21146621	-	-	-	+
#7: +77 kb	chr10: 21083548-21084092	+	+	-	-
#8: +78 kb	chr10: 21082562-21083509	+	-	-	+
#9: +94 kb	chr10: 21067144-21067673	+	+	-	-
#10: +106 kb	chr10: 21054704-21055207	-	+	+	+
#11: +112 kb	chr10: 21048611-21049218	-	-	-	+

*: For ChIP-seq peaks of Jurkat cells, positions were lifted over to mouse genome (mm10).

Supplementary Table 2. Primers used for RT-PCR.

Taqman Probes from Thermo Fisher

Genes	Probe ID
Actinb	Mm00607939_s1
Apoe	Mm01307193_g1
Cebpe	Mm02030363_s1
Csf1r	Mm01266652_m1
Csf3r	Mm00432735_m1
Fcer1a	Mm00438867_m1
Gata1	Mm01352636_m1
Gata2	Mm00492301_m1
Gfi1	Mm00515853_m1
Irf8	Mm00492567_m1
Jun	Mm00495062_s1
Myb	Mm00501741_m1
Prss34	Mm00617666_g1
Tal1	Mm01187033_m1

Probe sequences for intercalating dyes-based RT-PCR

Genes	Forward primers	Reverse primers
Actinb	ctaaggccaaccgtgaaaag	accagaggcatacagggaca
Myb	gaaagtgcctcaccagcaaggt	cgagctttcatggttgctggaag

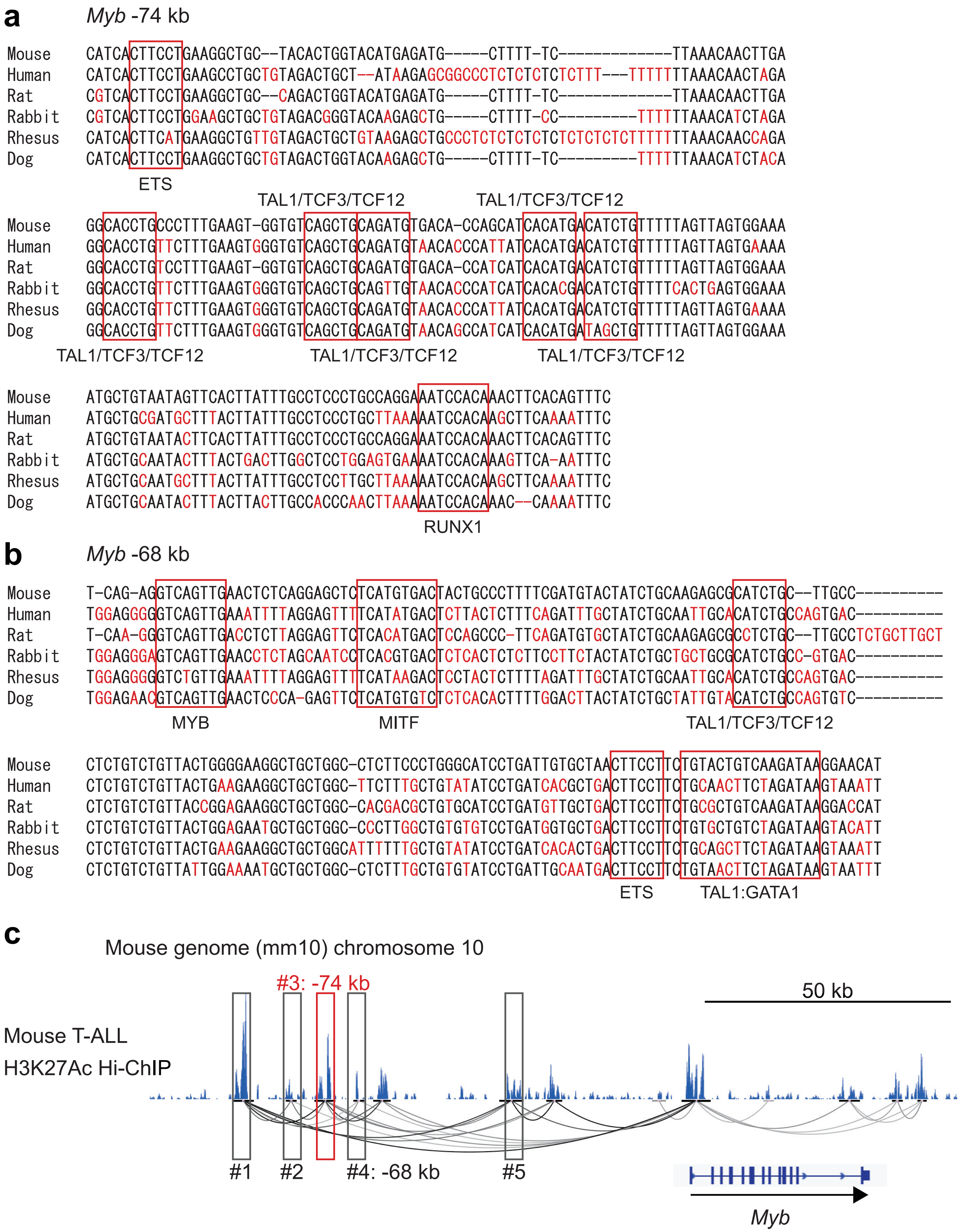

Supplementary Figure 1

Supplementary Figure 1. In silico analysis to identify cis-regulatory elements of *Myb*. (a and b) Sequence alignment of the core part of the *Myb* -74 kb element (a) and the *Myb* -68 kb element (b). Predicted consensus transcription factor binding sites are shown in red rectangles. Nucleotides not conserved are shown in red. Note that a compound binding motif of TAL1 and GATA1 is a TG 7 or 8 bp upstream of a WGATAA motif. (c) Re-analysis of our previously published histone H3 lysine 27 acetylation (H3K27Ac) Hi-ChIP data obtained from mouse T-ALL cells. Interactions among putative enhancer regions and the *Myb* promoter are shown. Candidate enhancer elements are shown in red or grey rectangles (#1 to #5).

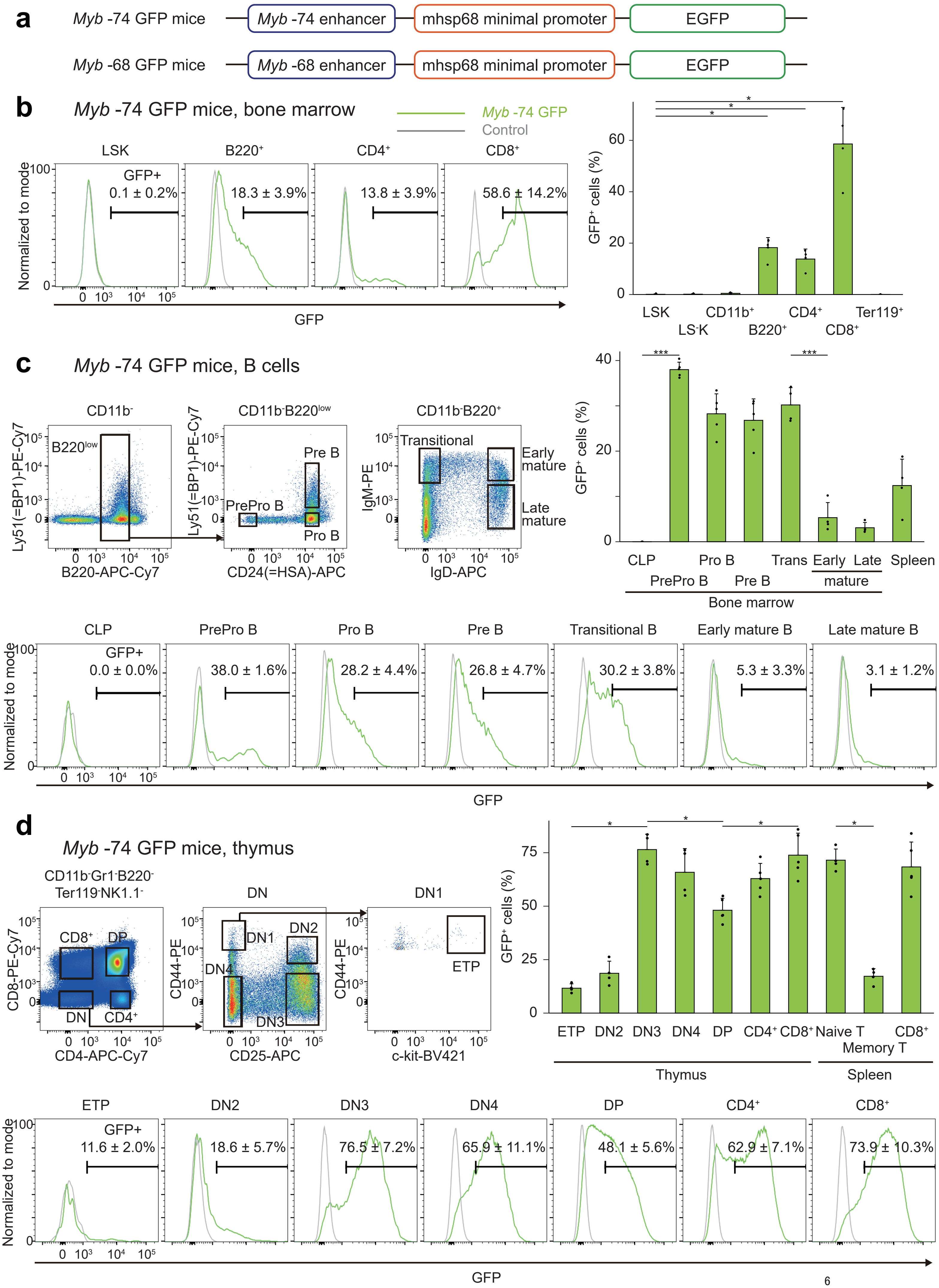

Supplementary Figure 2

Supplementary Figure 2. The *Myb* -74 enhancer is active in T and B cells in *Myb* -74 GFP transgenic mice. (a) A schema showing the constructs to generate transgenic enhanced GFP (EGFP) reporter mice, *Myb* -74 GFP mice (upper) and *Myb* -68 GFP mice (lower). The *Myb* -74 and -68 enhancers were cloned into the upstream of the mouse heatshock protein 68 (mhsp68) minimal promoter and EGFP. (b, c and d) GFP activity in bone marrow (b and c), thymus and spleen (d) of *Myb* -74 GFP mice. FACS gating strategies for sorting are shown in Supplementary Figures 12 (bone marrow), 13a (spleen), and 13b (thymus). Representative FACS plots of indicated cells from control (grey) and *Myb* -74 GFP mice (green) are shown. Numbers shown are mean percentages ± SD of GFP^+ cells in *Myb* -74 GFP mice. (b) n = 5 mice for LSK cells, LS^-K cells, $CD11b^+$ cells, and $B220^+$ cells, and n = 4 mice for others. $p = 8.0 \times 10^{-6}$ by one-way Welch's ANOVA. *$p < 0.05$ by the Games-Howell post hoc test. $p = 3.4 \times 10^{-3}$, 0.032, and 0.021 from left to right. (c) n = 5 mice for CLPs, and n = 4 mice for others. $p = 1.8 \times 10^{-16}$ by one-way ANOVA. Welch's ANOVA was unable to be performed because all values are zero in common lymphoid progenitors (CLP). ***$p < 0.001$ by the Games-Howell post hoc test. $p = 7.0 \times 10^{-6}$ between PrePro B and CLPs, and $p = 8.4 \times 10^{-4}$ between early mature B cells (Early) and transitional B cells (Trans). (d) n = 5 mice for double positive (DP) cells, $CD4^+$ cells, $CD8^+$ cells, and spleen $CD8^+$ cells, and n = 4 for others. $p = 1.4 \times 10^{-10}$ by one-way Welch's ANOVA. *$p < 0.05$ by the Games-Howell post hoc test. p = 0.0015, 0.012, 0.035, and 1.1×10^{-4} from left to right.

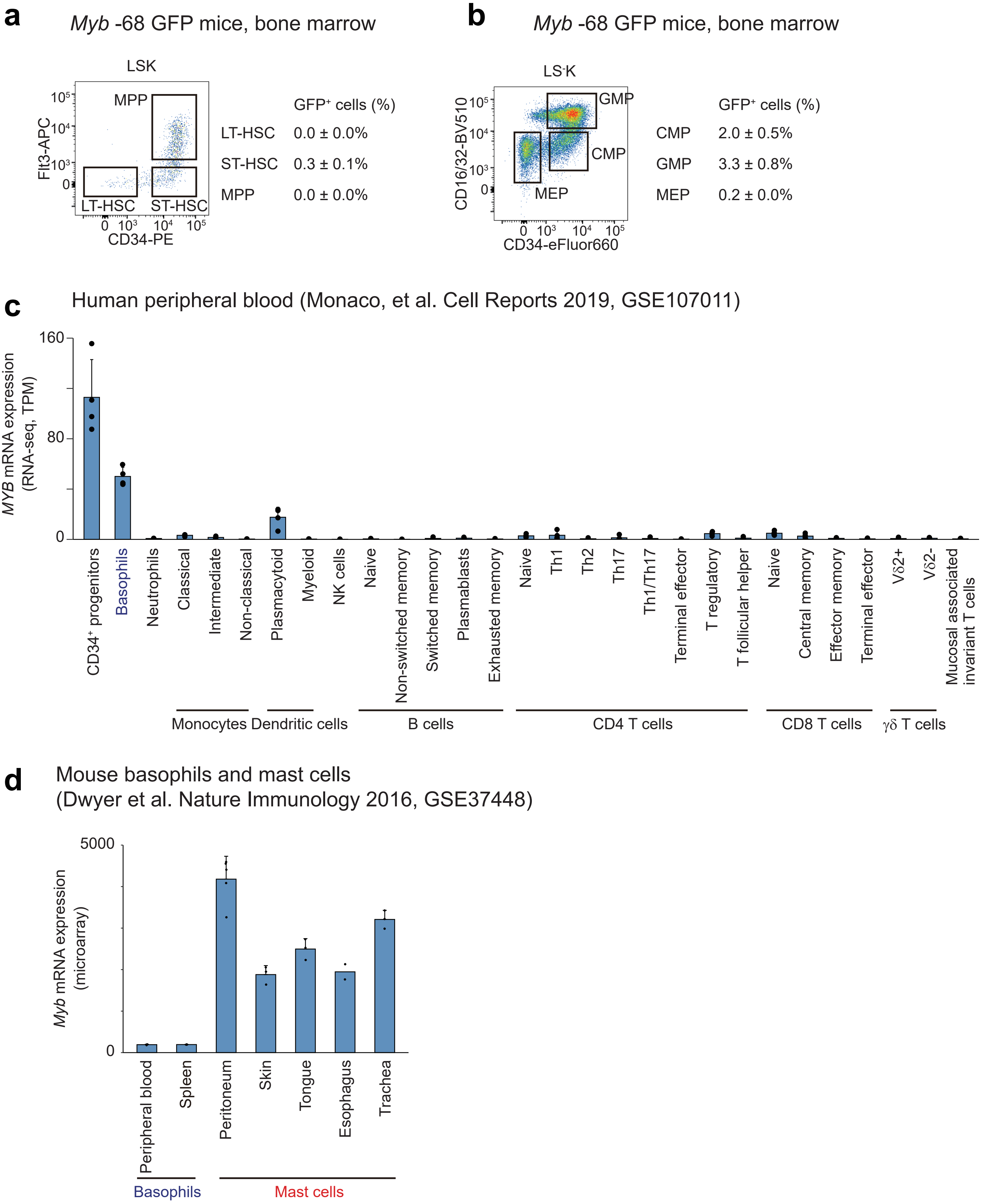

Supplementary Figure 3

Supplementary Figure 3. The *Myb* -68 enhancer is active in Ly6C^{-} GMPs and its function persists only in basophils and mast cells. (a and b) GFP activity in LSK cells (a) and LS^{-}K cells (b) of *Myb* -68 GFP mice. FACS gating strategies for sorting are shown in Supplementary Figures 12. Representative FACS plots are shown. Numbers shown are mean percentages ± SD of GFP^{+} cells. n = 3 mice. LT-HSC, long-term hematopoietic stem cells (HSC); ST-HSC, short-term HSCs; MPP, multipotent progenitors; CMP, common myeloid progenitor; GMP, granulocyte-macrophage progenitors; MEP, megakaryocyte-erythrocyte progenitors. (c) Analysis of publicly available RNA-seq data showing *MYB* mRNA expression in human peripheral blood. n = 2 for CD4^{+} terminal effector T cells, and n = 4 for others. $p = 4.9 \times 10^{-43}$ by one-way ANOVA. $p = 5.0 \times 10^{-9}$ by Welch's ANOVA. (d) Analysis of publicly available microarray data showing *Myb* mRNA expression in mouse basophils and mast cells. n = 5 for peritoneum mast cells, and n = 2 for esophagus mast cells, and n = 4 for others. $p = 2.7 \times 10^{-10}$ by one-way ANOVA.

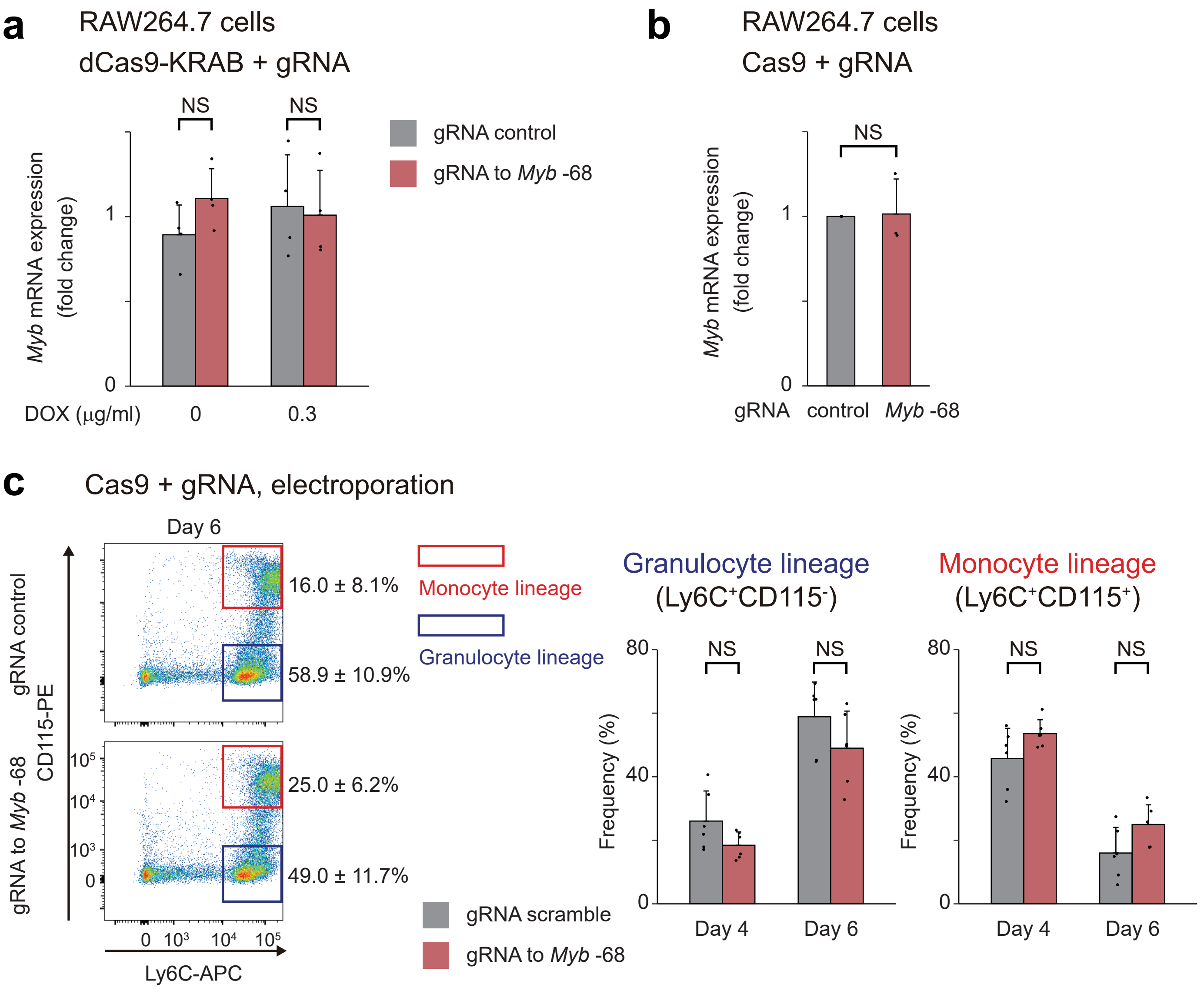

Supplementary Figure 4

Supplementary Figure 4. The *Myb* -68 enhancer regulates *Myb* mRNA expression only in basophils and mast cells. (a) Mouse macrophage-like RAW 264.7 cells were infected with lentivirus harboring dCas9-KRAB and DOX-inducible control gRNA (grey) or gRNA targeted for the *Myb* -68 enhancer (red). Infected cells were sorted and treated with doxycycline (0.3 ng/ml) for 72 hours, and then *Myb* mRNA expression was analyzed by qPCR. n = 4 independent experiments. Data are presented as mean values ± SD. p = 0.62 by one-way ANOVA. p = 0.59 and 0.99 from left to right by the Tukey-Kramer post hoc test. (b) Mouse macrophage-like RAW 264.7 cells were infected with lentivirus harboring Cas9 and DOX-inducible control gRNA (grey) or gRNA targeted for the *Myb* -68 enhancer (red). Cells were sorted and treated with doxycycline (1 ng/ml) for 72 hours. *Myb* mRNA expression was analyzed by qPCR (right). n = 3 independent experiments. Data are presented as mean values ± SD. p = 0.91 by two-tailed Student's t-test. (c) Cas9 protein and control gRNA (upper) or gRNA targeting the *Myb* -68 enhancer (lower) were delivered by electroporation into oligopotent $Ly6C^-$ GMPs harvested from wild-type bone marrow. FACS gating strategies for sorting are shown in Supplementary Figures 14a. Representative FACS plots 6 days after electroporation are shown. Numbers shown are mean percentages ± SD of granulocyte-lineage cells, defined as $Ly6C^+CD115^-$ (blue), and monocyte-lineage cells, defined as $Ly6C^+CD115^+$ (red). The percentages of granulocyte-lineage and monocyte-lineage cells are also shown in the right graph. n = 6 from 3 electroporation procedures. p = 0.10, 0.16, 0.10, and 0.06 from left to right by two-tailed Student's t-test without adjustments for multiple comparisons.

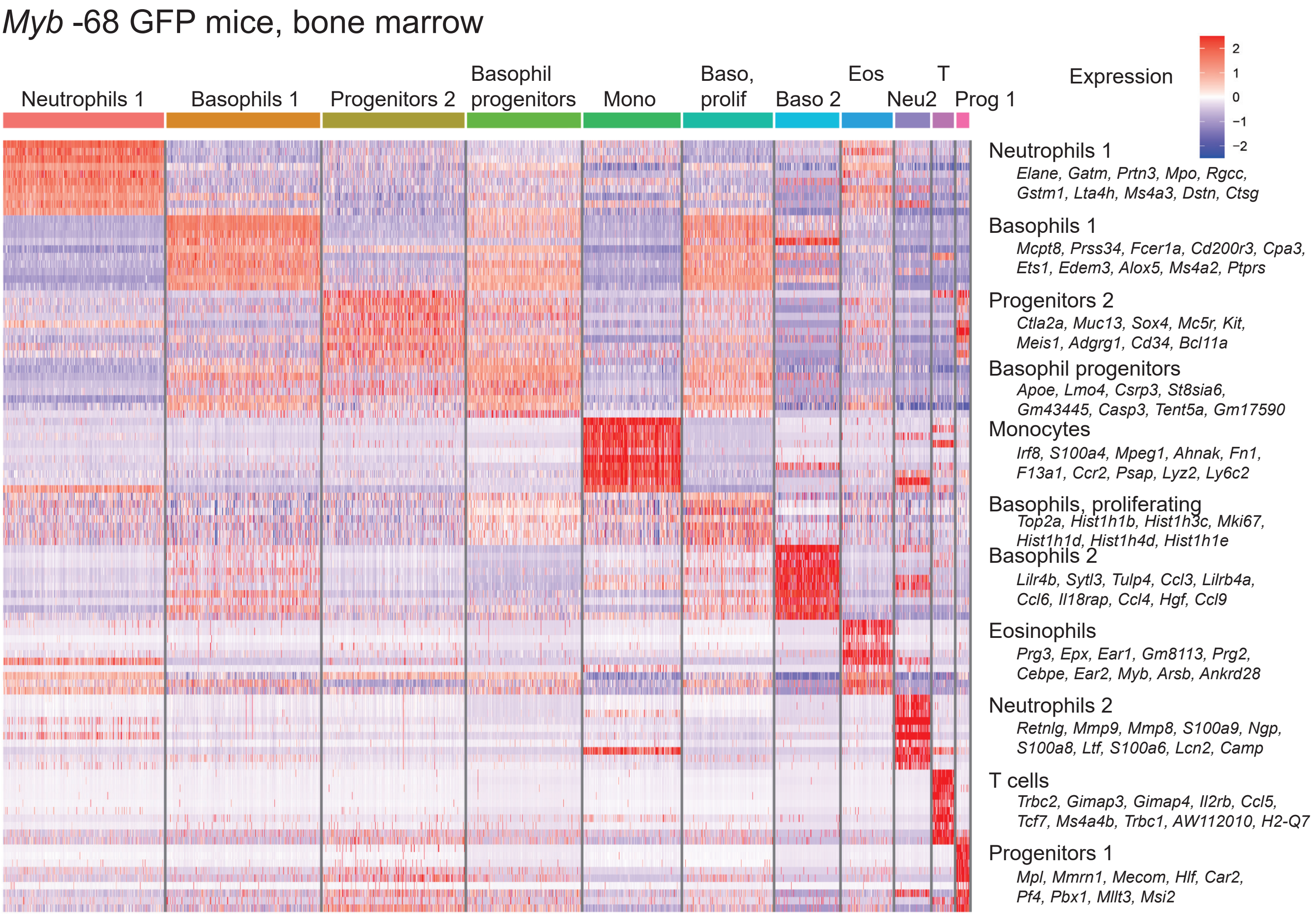

Supplementary Figure 5

Supplementary Figure 5. scRNA-seq analysis reveals an unperturbed in vivo differentiation trajectory of basophils. A heatmap of scRNA-seq of bone marrow GFP^+ cells in *Myb* -68 GFP mice, showing the top differentially expressed genes in each cluster.

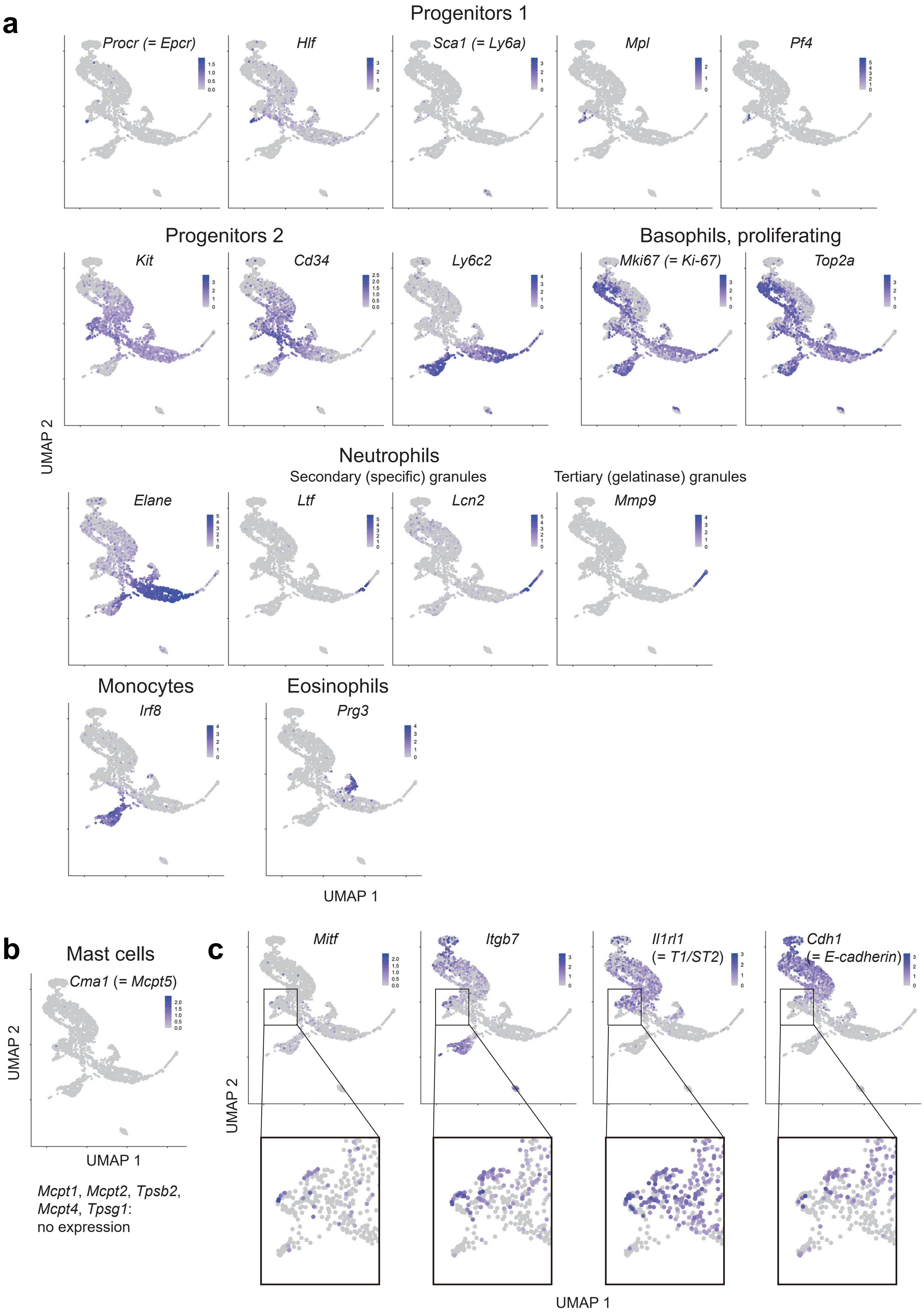

Supplementary Figure 6

Supplementary Figure 6. scRNA-seq analysis reveals an unperturbed in vivo differentiation trajectory of basophils. (a) UMAP projection of selected hallmark genes for each cluster. Note that neutrophil secondary (specific) granule genes, *Ltf* and *Lcn2*, and a tertiary (gelatinase) granule gene *Mmp9* are expressed only in the tip of the “Neutrophils 2” cluster, indicating that most neutrophil-lineage cells are immature. (b) UMAP projection of mast cell genes. Because *Mcpt1*, *Mcpt2*, *Tpsb2*, *Mcpt4*, and *Tpsg1* were not detected in any cells in our dataset, UMAP visualization of these genes is not available. (c) UMAP projection of basophil/mast cell progenitor marker genes. A part of the Progenitors 2 cluster was magnified in the bottom of each panel. In the magnified panels, cells were displayed in order of expression to prevent cells with high expression from getting buried.

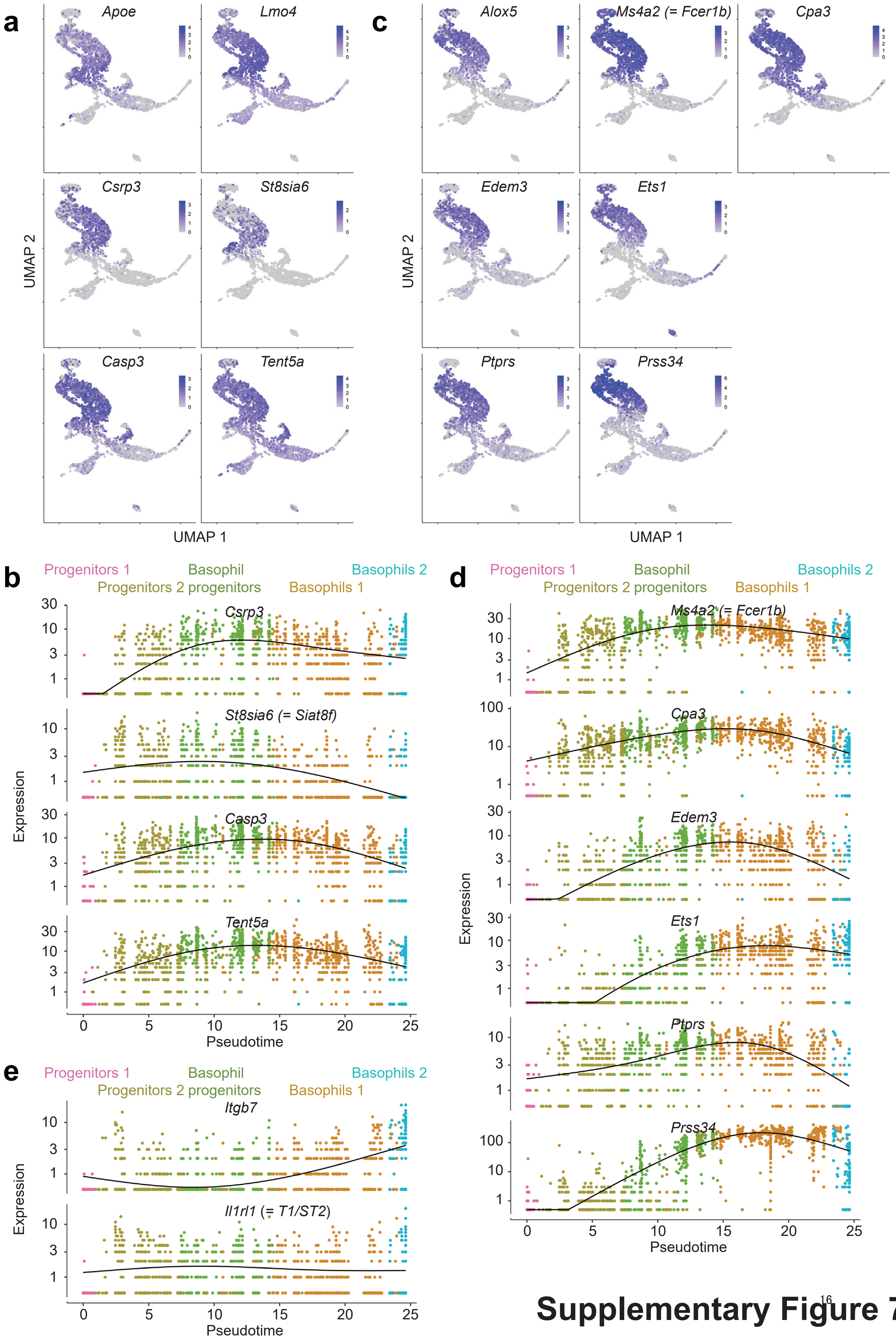

Supplementary Figure 7

Supplementary Figure 7. scRNA-seq analysis reveals an unperturbed in vivo differentiation trajectory of basophils. (a and c) UMAP projection of selected early (a) and late (c) basophil signature genes. (b, d and e) Pseudotime analysis for selected clusters: Progenitors 1 (pink), Progenitors 2 (dark yellow), Basophil progenitors (dark green), Basophils 1 (brown), and Basophils 2 (cyan). Expression levels of selected early (b) and late (d) basophil signature genes and previously reported progenitor marker genes (e) are shown.

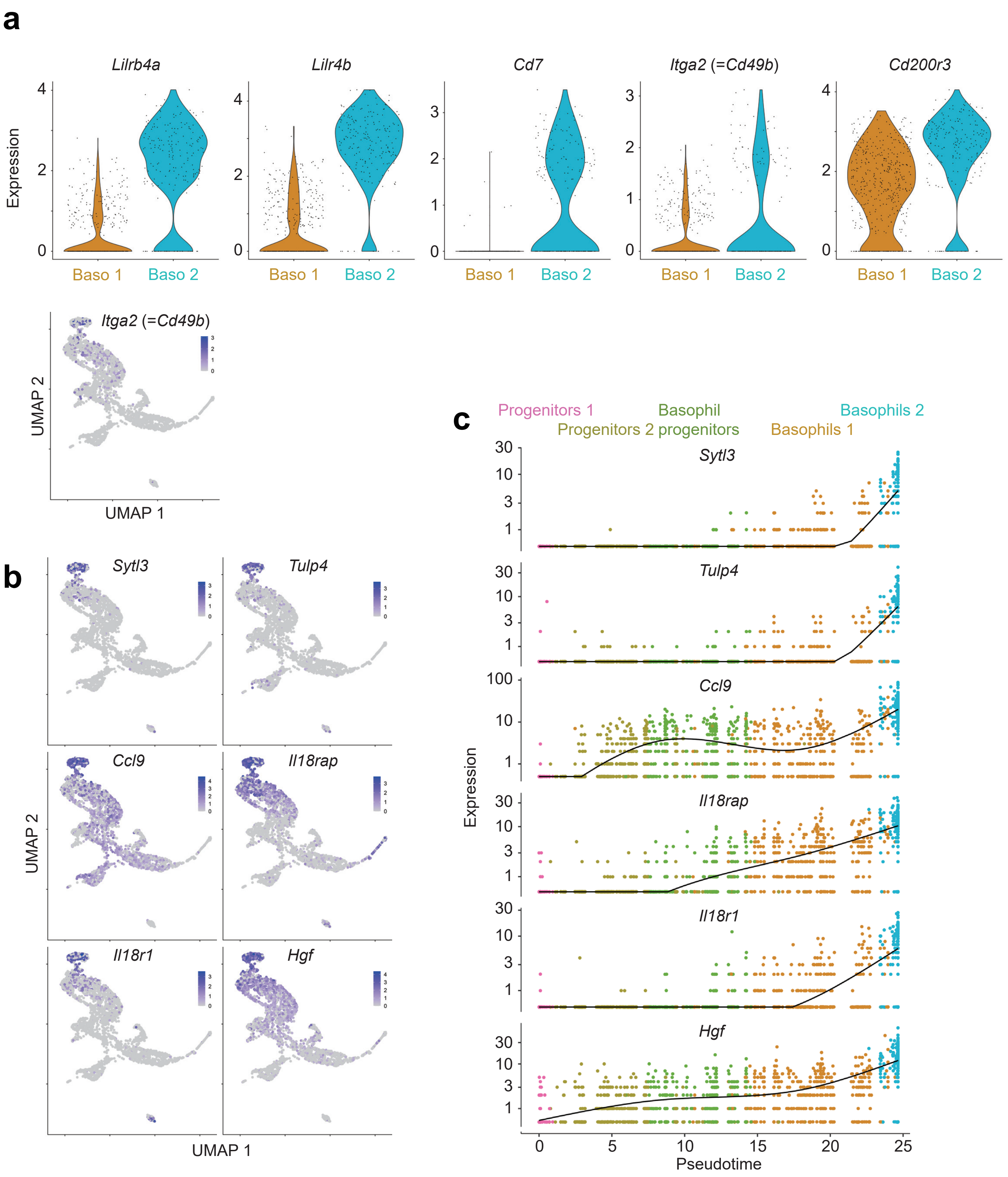

Supplementary Figure 8

Supplementary Figure 8. High-resolution fractionation of immature and mature basophils by scRNA-seq and FACS analysis. (a) Violin plots showing the difference of mRNA expression levels of selected basophil-lineage surface marker genes between Basophils 1 (Baso 1, brown) and Basophils 2 (Baso 2, cyan). The lower panel is UMAP projection of *Itga2*. (b) UMAP projection of selected Basophils 2 signature genes. (c) Pseudotime analysis for selected clusters: Progenitors 1 (pink), Progenitors 2 (dark yellow), Basophil progenitors (dark green), Basophils 1 (brown), and Basophils 2 (cyan). Expression levels of selected Basophils 2 signature genes are shown.

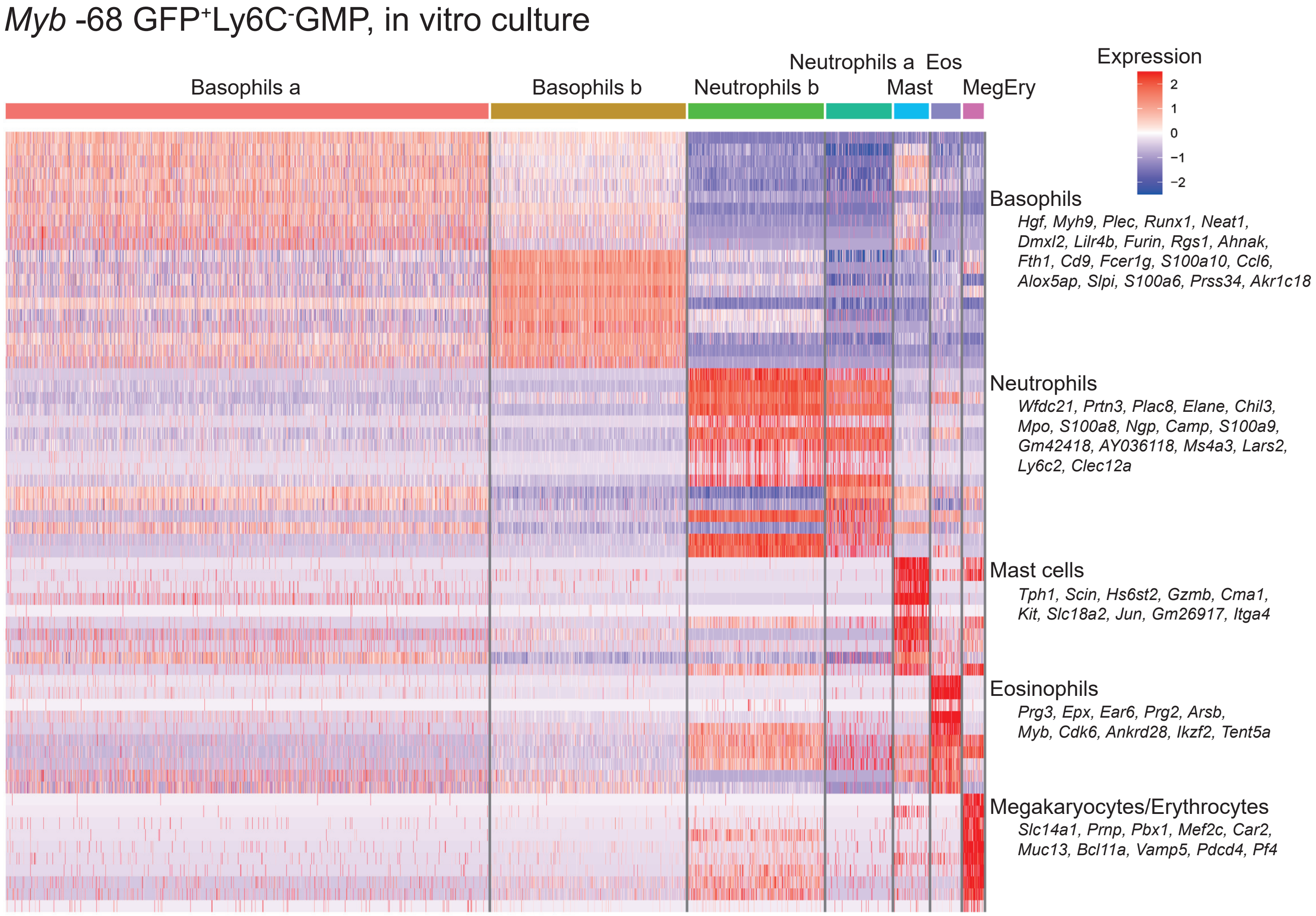

Supplementary Figure 9

Supplementary Figure 9. Integration of two scRNA-seq datasets identified bone marrow cells with a gene signature of immature mast cells. A heatmap of scRNA-seq of in vitro mast cell culture, showing the top differentially expressed genes in each cluster.

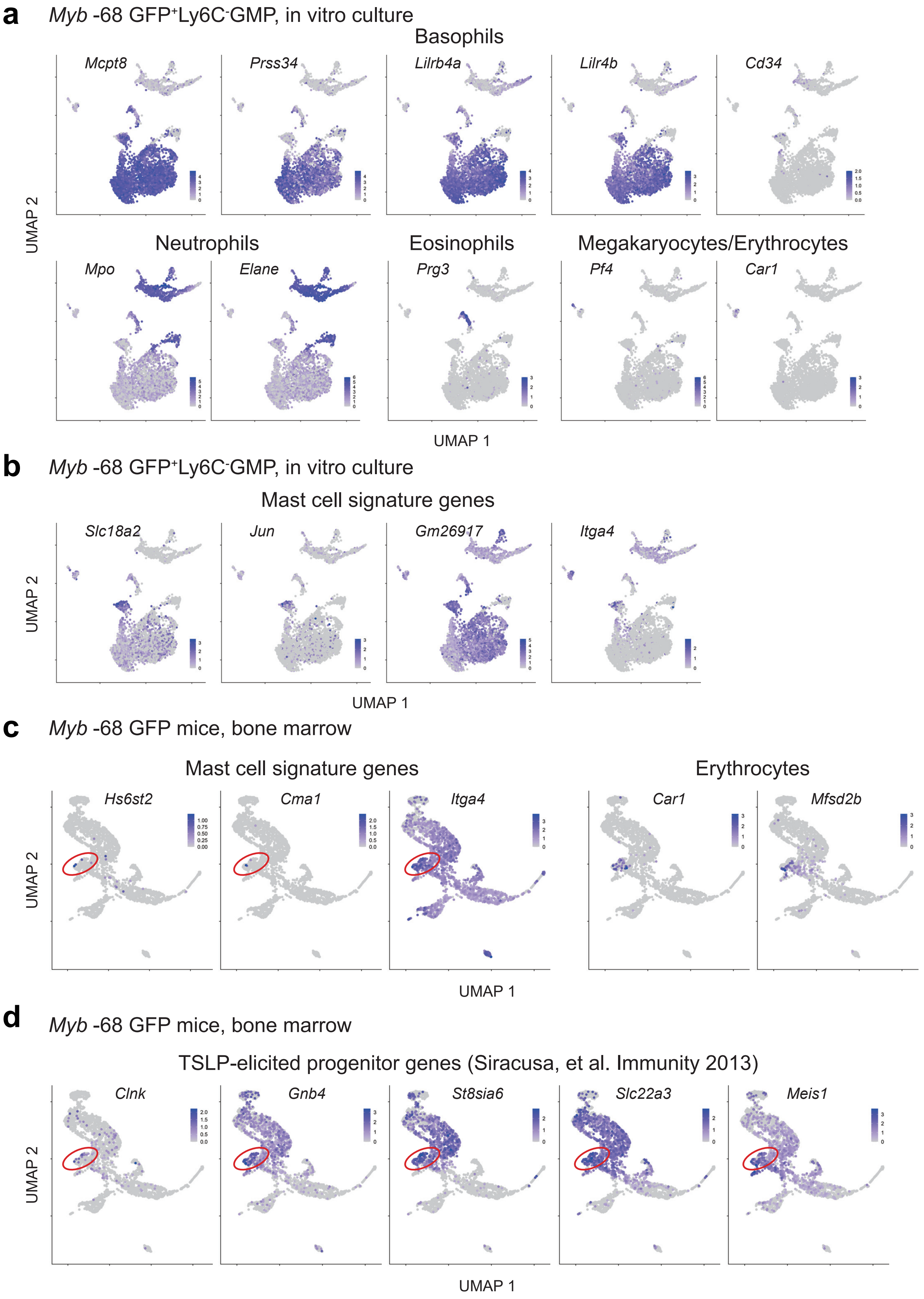

Supplementary Figure 10

Supplementary Figure 10. Integration of two scRNA-seq datasets identified bone marrow cells with a gene signature of immature mast cells. (a) UMAP projection of selected hallmark genes for each cluster in scRNA-seq of in vitro mast cell culture. (b) UMAP projection of mast cell signature genes in scRNA-seq of in vitro mast cell culture. (c and d) UMAP projection of mast cell signature genes (c, left), erythrocyte genes (c, right) and thymic stromal lymphopoietin (TSLP)-elicited progenitor genes (d) in scRNA-seq of bone marrow GFP^{+} cells in *Myb* -68 GFP mice. Cells with enrichment of the mast cell gene signature were shown in red. Cells were displayed in order of expression to prevent cells with high expression from getting buried.

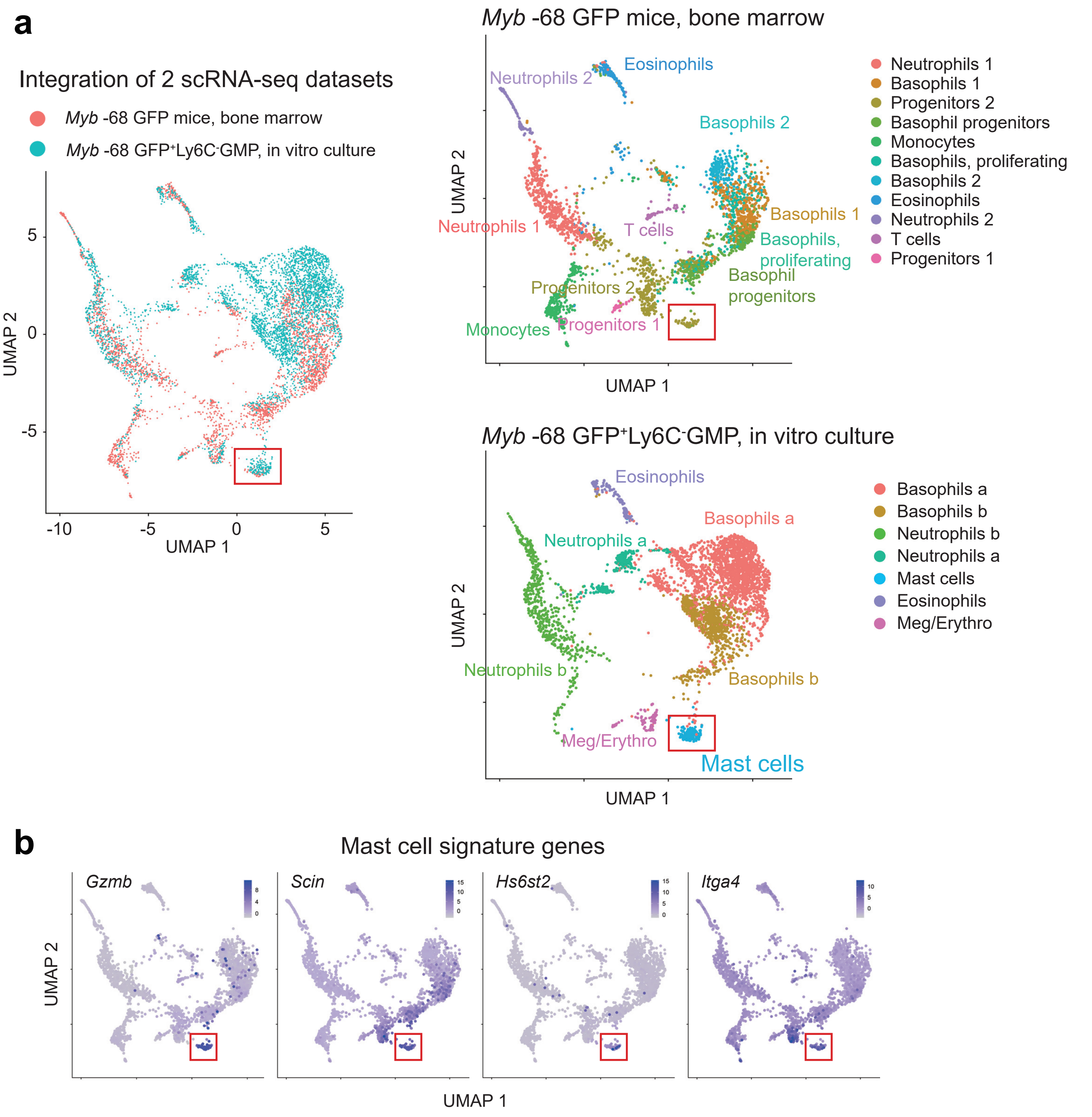

Supplementary Figure 11

Supplementary Figure 11. Integration of two scRNA-seq datasets identified bone marrow cells with a gene signature of immature mast cells. (a) The left panel demonstrates integration of scRNA-seq of bone marrow GFP$^+$ cells in *Myb* -68 GFP mice (red) and scRNA-seq of mast cell culture (blue). The right panels indicate the position of original clusters in each scRNA-seq data. (b) UMAP projection of mast cell signature genes. The location of the mast cell cluster identified in culture is shown in red rectangles. Cells were displayed in order of expression to prevent cells with high expression from getting buried.

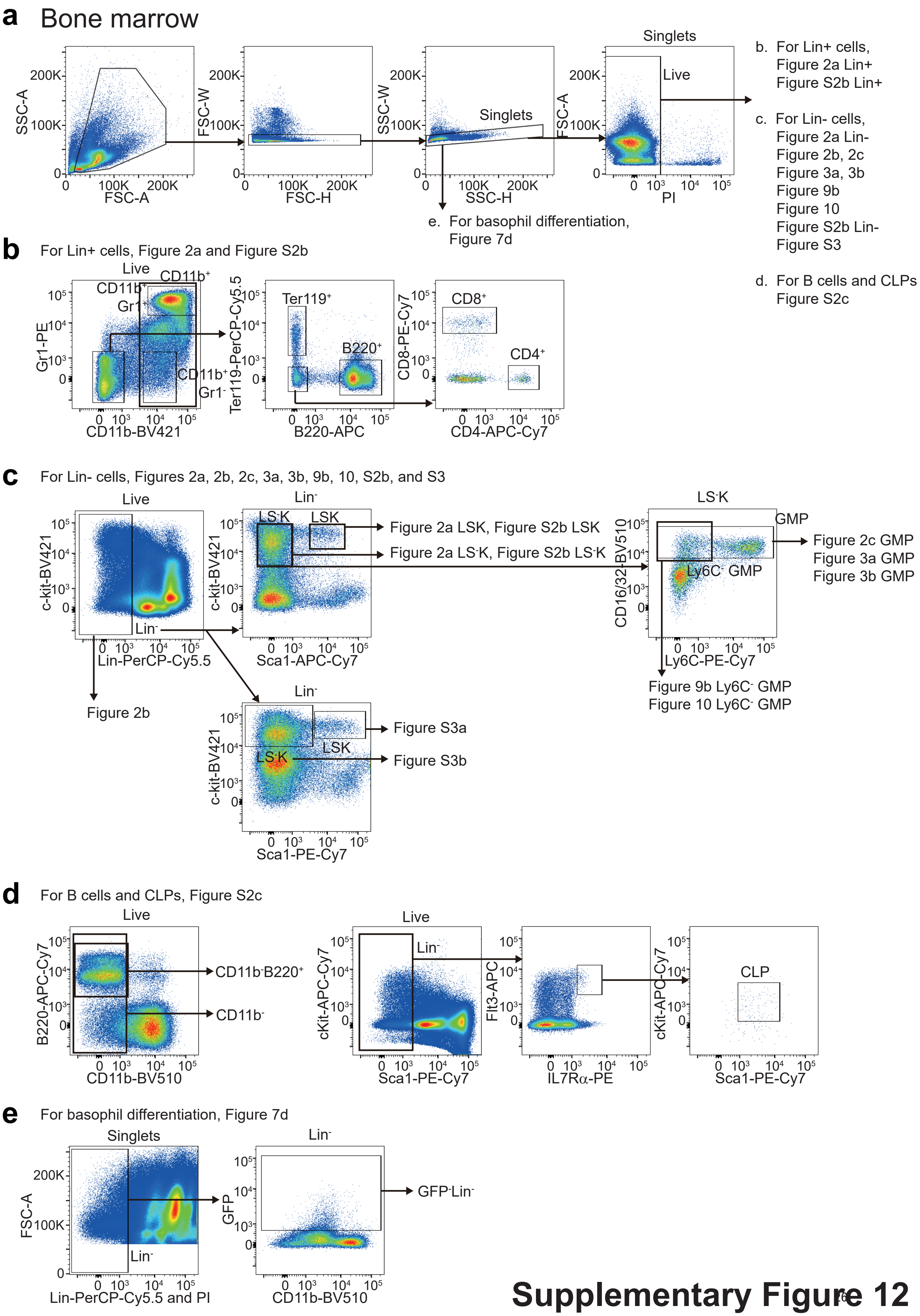

Supplementary Figure 12

Supplementary Figure 12. FACS gating strategies for bone marrow. (a) FACS gating strategies for bone marrow until singlets are selected. (b) FACS gating strategies to select lineage cells in Figure 2a and Supplementary Figure 2b. (c) FACS gating strategies to select Lin$^-$ cells in Figures 2a, 2b, 2c, 3a, 3b, 9b, and 10, and Supplementary Figures 2b and 3. (d) FACS gating strategies to select B cell-lineage cells and common lymphoid progenitors (CLP) in Supplementary Figure 2c. (e) FACS gating strategies for basophil differentiation in Figure 7d. Note that FACS gating strategies in Figure 9a are shown separately in Supplementary Figure 14b.

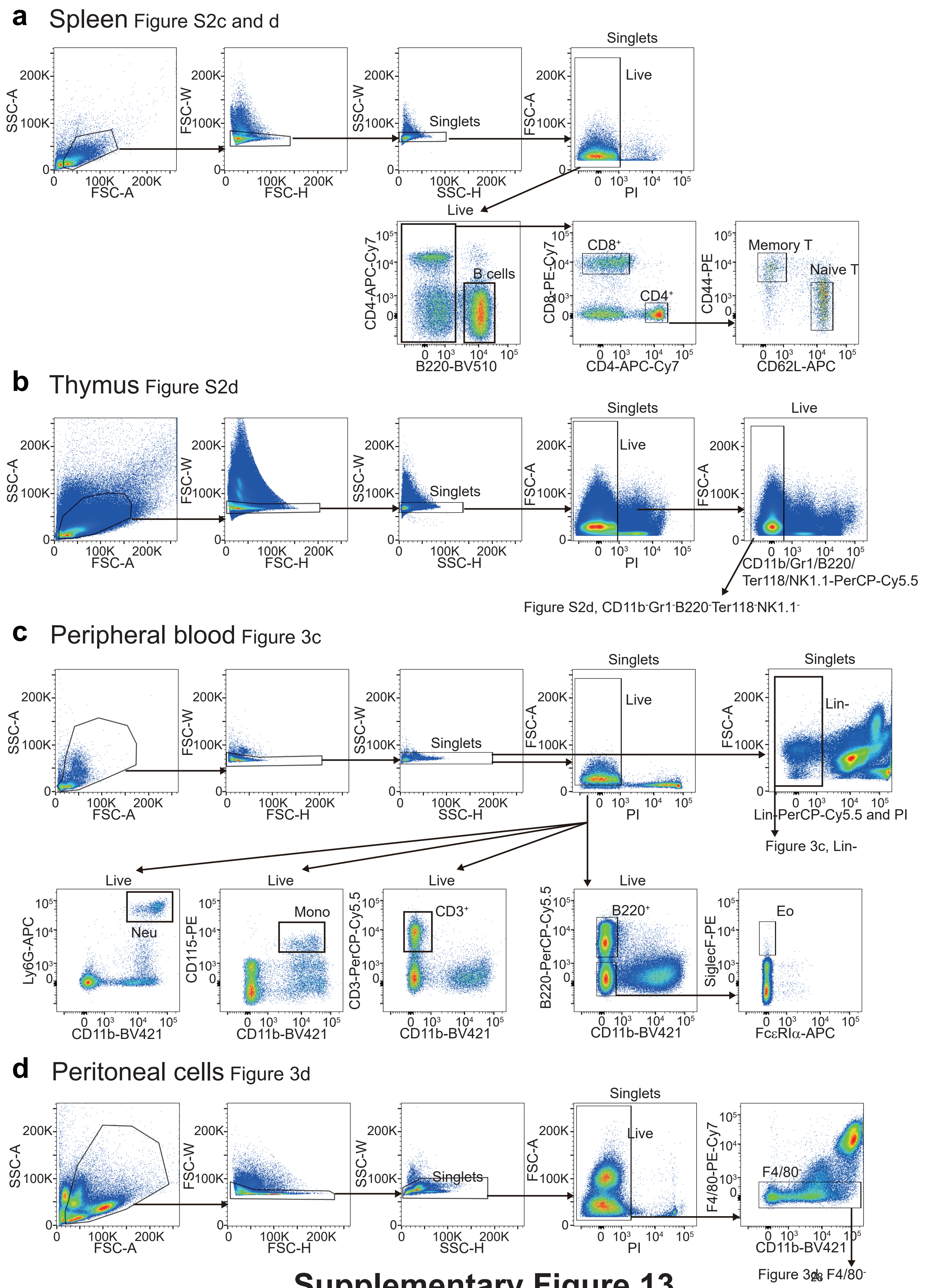

Supplementary Figure 13

Supplementary Figure 13. FACS gating strategies for spleen. thymus, peripheral blood, and peritoneal cells. (a) FACS gating strategies for spleen in Supplementary Figures 2c and 2d. (b) FACS gating strategies for thymus in Supplementary Figure 2d. (c) FACS gating strategies for peripheral blood in Figure 3c. (d) FACS gating strategies for peritoneal cells in Figure 3d.

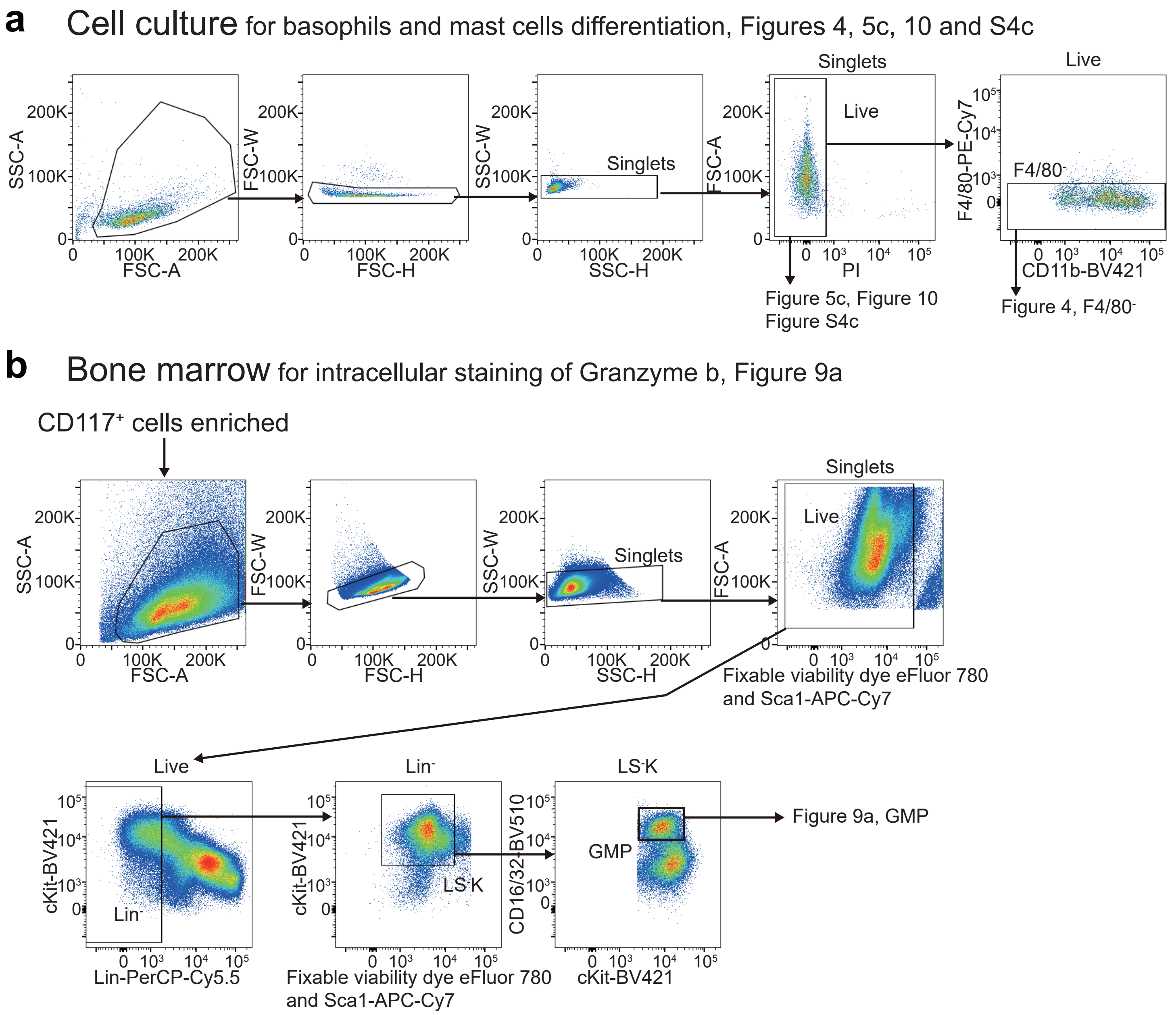

Supplementary Figure 14

Supplementary Figure 14. FACS gating strategies for cell culture and bone marrow for intracellular staining. (a) FACS gating strategies for basophil and mast cell culture in Figures 4, 5c, and 10 and Supplementary Figure 4c. (b) FACS gating strategies for bone marrow for intracellular staining of Granzyme b in Figure 9a. Note that dead cells and $Sca1^{+}$ cells were eliminated simultaneously in the same channel.
