# Supplementary material for: A Myb enhancer-guided analysis of basophil and mast cell differentiation
Source: Nat Commun. 2022 Nov 18;13:7064. doi: 10.1038/s41467-022-34906-1 (PMC9674656; doi:10.1038/s41467-022-34906-1)
Supplement: Supplementary file 2 — Reporting Summary [file 41467_2022_34906_MOESM2_ESM.pdf]

nature portfolio

Corresponding author(s): Takayoshi Matsumura

Last updated by author(s): Oct 11, 2022

Reporting Summary

Nature Portfolio wishes to improve the reproducibility of the work that we publish. This form provides structure for consistency and transparency in reporting. For further information on Nature Portfolio policies, see our Editorial Policies and the Editorial Policy Checklist.

Statistics

For all statistical analyses, confirm that the following items are present in the figure legend, table legend, main text, or Methods section.

n/a	Confirmed	
☐	☒	The exact sample size (n) for each experimental group/condition, given as a discrete number and unit of measurement
☐	☒	A statement on whether measurements were taken from distinct samples or whether the same sample was measured repeatedly
☐	☒	The statistical test(s) used AND whether they are one- or two-sided<br>*Only common tests should be described solely by name; describe more complex techniques in the Methods section.*
☒	☐	A description of all covariates tested
☐	☒	A description of any assumptions or corrections, such as tests of normality and adjustment for multiple comparisons
☐	☒	A full description of the statistical parameters including central tendency (e.g. means) or other basic estimates (e.g. regression coefficient) AND variation (e.g. standard deviation) or associated estimates of uncertainty (e.g. confidence intervals)
☐	☒	For null hypothesis testing, the test statistic (e.g. F, t, r) with confidence intervals, effect sizes, degrees of freedom and P value noted<br>*Give P values as exact values whenever suitable.*
☒	☐	For Bayesian analysis, information on the choice of priors and Markov chain Monte Carlo settings
☒	☐	For hierarchical and complex designs, identification of the appropriate level for tests and full reporting of outcomes
☒	☐	Estimates of effect sizes (e.g. Cohen's d, Pearson's r), indicating how they were calculated

Our web collection on statistics for biologists contains articles on many of the points above.

Software and code

Policy information about availability of computer code

Data collection	No software was used for data collection.

Data analysis	#1 single cell RNA-seq<br>Raw unique molecular identifier (UMI)-based data files were mapped against the mm10 reference genome, and mapped reads were counted using the Cell Ranger package (10x Genomics, version 6.0.0) with default parameters. The Seurat package (version 4.1.0) in R (version 4.1.1) was used to analyze the scRNA-seq data. Clusters were detected using FindClusters, and annotated based on feature genes. To mitigate the effects of cell cycle heterogeneity, cell-cycle scoring and regression were performed using CellCycleScoring and ScaleData. Differentially expressed genes were identified by running FindAllMarkers. The Monocle3 package (version 1.0.0) in R (version 4.1.1) was used to determine the pseudotime of basophil differentiation. Cells belonging to basophil lineage, namely Progenitors 1, Progenitors 2, Basophil progenitors, Basophils 1, and Basophils 2, were selected and used to construct single-cell trajectories. Gene set variation analysis (GSVA) was conducted by using the GSVA package (version 1.40.1) in R (version 4.1.1) and hallmark gene sets from the Molecular Signatures Database (MSigDB). Differentially enriched gene sets between Basophils 1 and Basophils 2 were determined and ranked by the limma package (version 3.48.3) in R (version 4.1.1). Two scRNA-seq datasets were integrated by running FindIntegrationAnchors and IntegrateData in the Seurat package (version 4.1.0).<br>#2. Analysis of previously published ChIP data.<br>For GSE22178, GSE29181, and ERA000161, reads that passed the quality filter step were mapped to the reference mouse or human genome sequence (mm10 or hg38) using Bowtie2 (version 2.4.2). Coverage tracks were generated by deepTools (version 3.5.1), and visualized by the Integrative Genomics Viewer (version 2.4.14). For GSE59636 and GSE48086, bigwig and bedgraph files deposited in GEO were visualized by the Integrative Genomics Viewer (version 2.4.14). Mouse T-ALL cells Hi-ChIP data was analyzed by Hi-C Pro (version 2.11.1) and hichipper (version 0.7.7).<br>#3. FACS data<br>FACS data were analyzed with the FlowJo analysis software (FlowJo, LLC, version 10.8.0).

For manuscripts utilizing custom algorithms or software that are central to the research but not yet described in published literature, software must be made available to editors and reviewers. We strongly encourage code deposition in a community repository (e.g. GitHub). See the Nature Portfolio guidelines for submitting code & software for further information.

Data

Policy information about availability of data

All manuscripts must include a data availability statement. This statement should provide the following information, where applicable:

- Accession codes, unique identifiers, or web links for publicly available datasets
- A description of any restrictions on data availability
- For clinical datasets or third party data, please ensure that the statement adheres to our policy

The scRNA-seq data generated in this study have been deposited in the Gene Expression Omnibus database: accession numbers GSE207688 (https://www.ncbi.nlm.nih.gov/geo/query/acc.cgi?acc=GSE207688) and GSE207689 (https://www.ncbi.nlm.nih.gov/geo/query/acc.cgi?acc=GSE207689). Previously published sequencing data (GSE22178 [https://www.ncbi.nlm.nih.gov/geo/query/acc.cgi?acc=GSE22178], GSE29181 [https://www.ncbi.nlm.nih.gov/geo/query/acc.cgi?acc=GSE29181], ERA000161 [https://www.ebi.ac.uk/ena/browser/view/ERA000161], GSE59636 [https://www.ncbi.nlm.nih.gov/geo/query/acc.cgi?acc=GSE59636], GSE48086 [https://www.ncbi.nlm.nih.gov/geo/query/acc.cgi?acc=GSE48086], and GSE115363 [https://www.ncbi.nlm.nih.gov/geo/query/acc.cgi?acc=GSE115363]) were available from each site. Reference mouse and human genome sequences (mm10 and hg38) are available from iGenomes (Illumina, https://support.illumina.com/sequencing/sequencing_software/igenome.html). Source data are provided with this paper. The data that support this study are available from the corresponding authors upon reasonable request.

Field-specific reporting

Please select the one below that is the best fit for your research. If you are not sure, read the appropriate sections before making your selection.

☒ Life sciences ☐ Behavioural & social sciences ☐ Ecological, evolutionary & environmental sciences

For a reference copy of the document with all sections, see nature.com/documents/nr-reporting-summary-flat.pdf

Life sciences study design

All studies must disclose on these points even when the disclosure is negative.

Sample size	No statistical method is used to predetermine sample size. Sample sizes were chosen based on expected phenotypes and previous experience with assay variability. Our previous experience related to this study can be found in reference 17.
Data exclusions	No data exclusions were performed.
Replication	All attempts of replication were successful. At least 2-3 experiments were performed independently.
Randomization	Mice and mouse bone marrow-derived cells were allocated into each group based on their genotyping results. For zebrafish embryos, embryos available on the day were randomly used, and no experimental groups were set.
Blinding	Blinding was unnecessary because all measured values in this study were objective, and no subjective values were measured. In addition, blinding was practically impossible because mice genotypes were obvious by raw FACS data without knowing their genotyping results in most cases.

Reporting for specific materials, systems and methods

We require information from authors about some types of materials, experimental systems and methods used in many studies. Here, indicate whether each material, system or method listed is relevant to your study. If you are not sure if a list item applies to your research, read the appropriate section before selecting a response.

Materials & experimental systems

n/a	Involved in the study
☐	☒ Antibodies
☐	☒ Eukaryotic cell lines
☒	☐ Palaeontology and archaeology
☐	☒ Animals and other organisms
☒	☐ Human research participants
☒	☐ Clinical data
☒	☐ Dual use research of concern

Methods

n/a	Involved in the study
☒	☐ ChIP-seq
☐	☒ Flow cytometry
☒	☐ MRI-based neuroimaging

Antibodies

Antibodies used

For immunocytochemistry, rabbit polyclonal anti-SCIN antibody (Novus, NBP1-31721) was used at 1:250 dilution.
All FACS antibodies were used at 1:100 dilution. B220 (RA3-6B2) (Biolegend, 103207/103223/103235/103239/103247, BD, 553092/552772), CD3e (145-2C11) (Biolegend, 100327), CD4 (RM4.5/GK1.5) (Biolegend, 100413/100540, eBioscience, 17-0041-81), CD8a (53-6.7) (Biolegend, 100714/100734, BD, 552877), CD11b (M1/70) (Biolegend, 101228/101235/101245), CD11c (N418) (Biolegend 117323/117327), CD16/CD32 (93) (Biolegend, 101333), CD24 (M1/69) (Biolegend, 101807/101814), CD25 (PC61) (Biolegend, 102012), CD34 (RAM34) (eBioscience, 50-0341-82), CD34 (SA376A4) (Biolegend, 152203/152207), CD41 (MWReg30) (BD, 558040), CD44 (IM7) (BD, 553134), CD49b (DX5) (Biolegend, 108919/10892, eBioscience, 17-5971-81), CD62L (MEL-14) (Biolegend, 104411), CD105 (MJ7/18) (Biolegend, 120409), CD115 (AFS98) (BD, 566839), CD135 (A2F10) (Biolegend, 135305/135313, eBioscience, 17-1351-82), CD117 (= c-kit) (2B8) (Biolegend, 105811/105823/105826/105827, BD, 558163), CD135 (A2F10) (Biolegend, 135305, eBioscience, 17-1351-82), CD150 (TC15-12F12.2) (Biolegend, 115904/115909/115914), F4/80 (BM8) (Biolegend 123113/123115/123132), FceR1a (MAR-1) (Biolegend 134307/134315/134318), Gr1 (RB6-8C5) (Biolegend, 108408/108428/108412), Granzyme b (QA16A02) (Biolegend, 372203), IgD (11-26c.2a) (Biolegend, 405713), IgM (RMM-1) (Biolegend, 406507), IL7Ra (A7R34) (eBioscience, 12-1271-82, Biolegend, 135021), LILRB4 (H1.1) (Biolegend, 144904), Ly6C (HK1.4) (Biolegend, 128011/128015/128017), Ly6G (1A8) (Biolegend, 127607/127613), Ly51 (6C3) (Biolegend, 108307/108313, eBioscience, 17-5891-80), NK1.1 (PK136) (Biolegend, 108727), Sca1 (D7) (eBioscience, 17-5981-83, Biolegend, 108114/108126/108129), Siglec-F (E50-2440) (BD, 552126/565526), and Ter119 (TER-119) (Biolegend, 116228).

Validation

Rabbit polyclonal anti-SCIN antibody (Novus, NBP1-31721) was validated for immunocytochemistry by Novus. All FACS antibodies are validated for FACS by each manufacturer. The spec sheet of each antibody to confirm validation is available from each manufacturer (https://www.biolegend.com, https://www.bdbiosciences.com, https://www.thermofisher.com, and https://www.novusbio.com).

Eukaryotic cell lines

Policy information about cell lines

Cell line source(s)

Mouse mast cell P815 cells and mouse macrophage-like RAW264.7 cells were provided by the RIKEN BRC through the National BioResource Project of the MEXT, Japan. HEK293T cells were purchased from Takara Bio.

Authentication

P815 cells were purchased in December 2021, and used within half a year. RAW264.7 cells were used only as negative controls. HEK293T cells were used only for lentivirus production. Thus no authentication was conducted.

Mycoplasma contamination

Cells were regularly tested for mycoplasma contamination to confirm they are negative by using TaKaRa PCR Mycoplasma Detection Set.

Commonly misidentified lines
(See ICLAC register)

None

Animals and other organisms

Policy information about studies involving animals; ARRIVE guidelines recommended for reporting animal research

Laboratory animals

One-cell-stage embryos of the wild-type zebrafish (Danio rerio) AB line was used. Zebrafish embryo sex was not examined. Each construct was injected more than 100 eggs in one experiment, and at least two independent experiments were conducted for each construct.
Myb -74 kb and -68 kb enhancer element-EGFP transgenic mice on a C57BL/6 background were maintained as heterozygotes under specific pathogen-free conditions in a 12/12-hour light/dark cycle with food and water provided ad libitum. The room temperature for mice was between 20°C and 26°C, and the relative humidity was kept at between 30% and 70%. 8 to 16 weeks old, littermate-, age- and gender-matched mice were used. Thus, both male and female mice were used.

Wild animals	The study does not involve wild animals.
Field-collected samples	The study did not involve samples collected from the field.
Ethics oversight	All animal procedures were approved by the Institutional Animal Care Use Committee of the National University of Singapore (BR13-6034, BR17-1489, BR21-1070, R13-6200, R17-1512, and R21-1073) and were performed according to their recommendations.

Note that full information on the approval of the study protocol must also be provided in the manuscript.

Flow Cytometry

Plots

Confirm that:

- [x] The axis labels state the marker and fluorochrome used (e.g. CD4-FITC).
- [x] The axis scales are clearly visible. Include numbers along axes only for bottom left plot of group (a 'group' is an analysis of identical markers).
- [x] All plots are contour plots with outliers or pseudocolor plots.
- [x] A numerical value for number of cells or percentage (with statistics) is provided.

Methodology

Sample preparation	Bone marrow cells were harvested from femurs, tibias, and the spine of 2- to 4-month-old age- and sex-matched mice. The cells were dissociated to a single-cell suspension by filtering through a 70-μm nylon mesh. To analyze peripheral blood cells, 1.2% dextran in PBS was added to blood to sediment erythrocytes for 45 min at room temperature, and then the leukocyte-rich plasma above the sedimented erythrocytes was used. Cells were Fc-blocked and stained with anti-mouse primary antibodies for 60 min. All antibodies were purchased from Thermo Fisher, BD Biosciences or BioLegend. For sorting, cKit+ cells were pre-enriched with the CD117 MicroBeads and the MACS LS columns (Miltenyi Biotec).
Instrument	Cells were analyzed or sorted using LSRII and FACS Aria II cytometers (BD Biosciences).
Software	Subsequent data analyses were performed with the FlowJo analysis software (FlowJo, version 10.8.0, LLC).
Cell population abundance	The abundance of relevant cell groups were in general consistent with previous publications (ex. Lin-/Live cells = 5%, LSK/Lin- = 5%). In post-sort fractions, the purity was >90%, determined by FACS analysis of post-sort samples.
Gating strategy	For all analyses, debris was excluded by FSC-SSC plots, and singlets were selected. PI staining was used to exclude dead cells. FACS gating strategies for all figures were provided in Supplemental Figures 12 to 14.

- [x] Tick this box to confirm that a figure exemplifying the gating strategy is provided in the Supplementary Information.
